# Gestational weight gain charts: results from the Brazilian Maternal and Child Nutrition Consortium

**DOI:** 10.1093/ajcn/nqaa402

**Published:** 2021-03-19

**Authors:** Gilberto Kac, Thaís R B Carilho, Kathleen M Rasmussen, Michael E Reichenheim, Dayana R Farias, Jennifer A Hutcheon, Brazilian Maternal and Child Nutrition Consortium, Adauto Emmerich Oliveira, Ana Paula Esteves-Pereira, Ana Paula Sayuri Sato, Antônio Augusto Moura da Silva, Caroline de Barros Gomes, Claudia Leite de Moraes, Claudia Saunders, Daniela da Silva Rocha, Dayana Rodrigues Farias, Denise Petrucci Gigante, Edson Theodoro dos Santos Neto, Elisa Maria de Aquino Lacerda, Elizabeth Fujimori, Eric O Ohuma, Fernanda Garanhani Surita, Gilberto Kac, Isabel Oliveira Bierhals, Jane de Carlos Santana Capelli, José Guilherme Cecatti, Juliana dos Santos Vaz, Juraci Almeida Cesar, Marco Fábio Mastroeni, Maria Antonieta de Barros Leite Carvalhaes, Maria do Carmo Leal, Mariângela Freitas da Silveira, Marlos Rodrigues Domingues, Mayra Pacheco Fernandes, Michael Eduardo Reichenheim, Michele Drehmer, Mônica de Araújo Batalha, Mylena Maciel Gonzalez, Nathalia Cristina de Freitas-Costa, Patrícia de Carvalho Padilha, Renato Teixeira Souza, Rosângela Fernandes Lucena Batista, Silmara Salete de Barros Silva Mastroeni, Silvana Granado Nogueira da Gama, Silvia Regina Dias Medici Saldiva, Simone Seixas da Cruz, Sirlei Siani Morais, Thais Rangel Bousquet Carrilho

**Affiliations:** Nutritional Epidemiology Observatory, Josué de Castro Nutrition Institute, Federal University of Rio de Janeiro, Rio de Janeiro, RJ, Brazil; Nutritional Epidemiology Observatory, Josué de Castro Nutrition Institute, Federal University of Rio de Janeiro, Rio de Janeiro, RJ, Brazil; Division of Nutritional Sciences, Cornell University, Ithaca, NY, USA; Department of Epidemiology, Institute of Social Medicine, Rio de Janeiro State University, Rio de Janeiro, RJ, Brazil; Nutritional Epidemiology Observatory, Josué de Castro Nutrition Institute, Federal University of Rio de Janeiro, Rio de Janeiro, RJ, Brazil; Department of Obstetrics and Gynaecology, University of British Columbia, Faculty of Medicine, Vancouver, BC, Canada; Federal University of Espírito Santo; Oswald Cruz Foundation; University of São Paulo; Federal University of Maranhão; Julio de Mesquita Filho Paulista State University; Rio de Janeiro State University; Federal University of Rio de Janeiro; Federal University of Bahia; Federal University of Rio de Janeiro; Federal University of Pelotas; Federal University of Espírito Santo; Federal University of Rio de Janeiro; University of São Paulo; London School of Hygiene & Tropical Medicine; University of Campinas; Federal University of Rio de Janeiro; Federal University of Pelotas; Federal University of Rio de Janeiro; University of Campinas; Federal University of Pelotas; Federal University of Rio Grande; University of Joinville Region; Julio de Mesquita Filho Paulista State University; Oswaldo Cruz Foundation; Federal University of Pelotas; Federal University of Pelotas; Federal University of Pelotas; Rio de Janeiro State University; Federal University of Rio Grande do Sul; Federal University of Rio de Janeiro; Federal University of Rio de Janeiro; Federal University of Rio de Janeiro; Federal University of Rio de Janeiro; University of Campinas; Federal Univesity of Maranhão; University of Joinville Region; Oswaldo Cruz Foundation; University of São Paulo; Federal University of Recôncavo da Bahia; University of Campinas; Federal University of Rio de Janeiro

**Keywords:** gestational weight gain, pregnancy, reference standards, primary health care, weight gain, gestation

## Abstract

**Background:**

Monitoring gestational weight gain (GWG) is fundamental to ensure a successful pregnancy for the mother and the offspring. There are several international GWG charts, but just a few for low- and middle-income countries.

**Objectives:**

To construct GWG charts according to pre-pregnancy BMI for Brazilian women.

**Methods:**

This is an individual patient data analysis using the Brazilian Maternal and Child Nutrition Consortium data, comprising 21 cohort studies. External validation was performed using “Birth in Brazil,” a nationwide study. We selected adult women with singleton pregnancies who were free of infectious and chronic diseases, gestational diabetes, and hypertensive disorders; who delivered a live birth at term; and whose children were adequate for gestational age, and with a birth weight between 2500–4000 g. Maternal self-reported pre-pregnancy weight and weight measured between 10–40 weeks of gestation were used to calculate GWG. Generalized Additive Models for Location, Scale and Shape were fitted to create GWG charts according to gestational age, stratified by pre-pregnancy BMI.

**Results:**

The cohort included 7086 women with 29,323 weight gain measurements to construct the charts and 4711 women with 31,052 measurements in the external validation. The predicted medians for GWG at 40 weeks, according to pre-pregnancy BMI, were: underweight, 14.1 kg (IQR, 10.8–17.5 kg); normal weight, 13.8 kg (IQR, 10.7–17.2 kg); overweight, 12.1 kg (IQR, 8.5–15.7 kg); obesity, 8.9 kg (IQR, 4.8–13.2 kg). The 10^th^, 25^th^, 50^th^, 75^th^, and 90^th^ percentiles were estimated. Results for internal and external validation showed that the percentages below the selected percentiles were close to those expected.

**Conclusions:**

The charts proposed provide a description of GWG patterns according to gestational age and pre-pregnancy BMI among healthy Brazilian women with good neonatal outcomes. The external validation indicates that this new tool can be used to monitor GWG in the primary health-care setting and to test potential recommended values.

## Introduction

Monitoring gestational weight gain (GWG) during pregnancy and developing proper recommendations on optimal weight gain are useful strategies to prevent the occurrence of adverse outcomes for both the mother and the child, such as small/large for gestational age (SGA/LGA), cesarean delivery and postpartum weight retention (1–3). GWG recommendations must consider the trade-off between risks that increase with high weight gain and those that increase with low weight gain (4, 5). Several local and international charts for monitoring GWG have been developed (6–10). However, some of them were created for women from high-income countries, who may have a GWG pattern that differs from that of women from low- and middle-income countries (LMICs). Appropriate tools to monitor weight or GWG in these countries are scarce. A few initiatives were developed for some countries, but with important limitations, such as using a small sample size, non-representative population, and not considering pre-pregnancy BMI (11–13).

Pre-pregnancy BMI is a strong predictor of GWG and, since 1990, recommendations of weight gain during pregnancy have differed according to a woman's BMI category (14, 15). GWG trajectories among women from different BMI categories tend to be different and, more importantly, pre-pregnancy BMI modifies the association between pregnancy weight gain and adverse outcomes. Thus, a different amount of GWG is recommended for each BMI category (15).

Brazil has never developed its own GWG recommendations for its diverse population. Since the 1980s, the Ministry of Health has incorporated charts and recommendations developed for other countries in the national public health system (11, 14–17). Currently, Atalah et al. (17) BMI charts, combined with the 2009 US Institute of Medicine (IOM) GWG recommendations (15), are in place. A study conducted in 2009 revealed that Atalah et al. (17) charts were inadequate to predict the occurrence of low birth weight, SGA, and LGA newborns (18); thus, the system used to monitor GWG in Brazil and other Latin American countries has low ability to predict the occurrence of neonatal outcomes. In addition, it has been shown that this tool can even classify excessive weight as “normal,” potentially contributing to the obesity epidemic (18).

In 2016, the International Fetal and Newborn Growth Consortium for the 21^st^ Century (INTERGROWTH-21^st^) published GWG standards with data from 8 countries, including Brazil (8). These standards have the potential to replace the current system adopted in Brazil, as they were derived from a highly prescribed population, included a good sample size, and pertained to 8 diverse populations. However, they are limited to women classified as normal weight and relied on a weight measured between 9–14 gestational weeks for GWG calculation, which decreases their utility for monitoring weight gain in the first trimester (the charts start at the 14^th^ gestational week). Because charts for all BMI categories are needed and a large proportion of women do not have a measure of weight collected during the first trimester (19, 20), incorporating the INTERGROWTH-21^st^ standards in the public health-care system in Brazil was not a practical option.

To address the limitations of the current monitoring system and offer a pragmatic tool that considers the reality of the country, we aimed to construct new GWG charts according to pre-pregnancy BMI for Brazilian women that could be adopted in the Brazilian primary health-care system and even by other LMICs with similar socio-demographic and nutritional profiles.

## Methods

### Study design and sample

This study uses data from the Brazilian Maternal and Child Nutrition Consortium (BMCNC), which combines individual patient data with repeated measurements of weight during pregnancy from 21 Brazilian studies conducted between 1990–2018. Details of the cohort creation and steps taken to harmonize key variables, such as gestational age, weight, and self-reported weight have been reported elsewhere (21).

The present study was restricted to women with singleton pregnancies, aged ≥18 years, who were free of infectious and chronic diseases (except obesity), without pregnancy complications of gestational diabetes or hypertensive disorders, and who delivered a live-born infant. We excluded women who did not have at least 1 measurement of weight during pregnancy or had no information on pre-pregnancy BMI. Outliers of weight and weight gain were removed from this analysis using previously described methods (21).

We further excluded pregnancies that delivered preterm (<37 weeks), SGA or LGA infants (<10^th^ and >90^th^ percentiles of the INTERGROWTH-21^st^ sex-specific neonatal charts, respectively) (22), or delivered infants with low birth weight (weight < 2500 g) or macrosomia (weight > 4000 g). We limited the charts to measurements between 10–40 weeks of pregnancy to ensure our estimates had reasonable statistical precision. Only approximately 7% of the measurements were taken outside this interval (369 women; 3514 measurements), and these were removed from the analyses.

### Study variables

Pregnancy weight gain was defined as the difference between the weight measured in each pregnancy visit and the self-reported pre-pregnancy weight. In a previous validation study, we found good agreement between self-reported weight and the weight measured in the first trimester among women with both values available, especially if the latter was measured up to the first 30–45 days of pregnancy (19). However, because a measurement in this restricted time frame is rarely available to antenatal care providers in Brazil, the use of self-reported pre-pregnancy weight is a pragmatic choice.

Pre-pregnancy BMI was calculated based on the self-reported pre-pregnancy weight (kg) and height (m^2^) measured in each study. Pre-pregnancy BMI (kg/m^2^) was classified according to the WHO cutoffs as underweight (<18.5), normal weight (≥18.5 and <25.0), overweight (≥25.0 and <30.0), or obesity (≥30.0) (23). Gestational age in each visit was determined based on ultrasound performed up to 24 weeks of pregnancy. If the exam was performed later or was not available, the last menstrual period date was used. Birth weight and length, mode of delivery, and maternal sociodemographic characteristics were used to describe the sample and obtained from the questionnaires of the original studies or medical records (21).

### Ethics

The Research Ethics Committee of the Rio de Janeiro Federal University Maternity Teaching Hospital approved this project (protocol number: 85914318.2.0000.5275). All analyses were conducted with deidentified data, and all incorporated studies from the BMCNC were individually approved by their own institutional research ethics committees and were conducted in accordance with the principles of the Declaration of Helsinki. The Birth in Brazil study was also approved by the Research Ethics Committee from the National School of Public Health (Oswaldo Cruz Foundation, Ministry of Health, opinion no. 92/10), and all women signed a Term of Free and Informed Consent.

### Statistical analysis

Characteristics of the study population were described using medians and IQRs for continuous variables and absolute (*n*) and relative (%) frequencies for categorical ones. Kruskal-Wallis and chi-squared tests were performed to compare the medians and frequencies of the selected variables according to pre-pregnancy BMI. Several different modeling strategies were considered for the construction of the charts. We documented our process of choosing our final model in **Supplementary Methods 1**. Briefly, we tested linear mixed models, fractional polynomials (incorporating or not incorporating clusters of individuals), restricted cubic splines, a combination of multilevel models (2-level random intercept and slope) and fractional polynomials, and Generalized Additive Models for Location, Scale, and Shape (GAMLSS). Several criteria guided the selection of the best model, including diagnostic measures and a comparison of the percentages of observed measurements below and above selected percentiles on the charts (3^rd^, 10^th^, 25^th^, 50^th^, 75^th^, 90^th^, and 97^th^). All models were stratified by pre-pregnancy BMI.

GAMLSS were used to produce the final BMI-specific charts. These models best described the distribution of GWG in each gestational age, which is the main goal when creating such charts. The use of GAMLSS allows modeling up to 4 parameters of the GWG distribution, i.e, mu (mean), sigma (SD), nu (or lambda, asymmetry), and tau (kurtosis). The LMS (lambda, mu, sigma) function, which is available in the GAMLSS package in R software, was used. This functions adjusts LMS [lambda, mu, sigma, the 3 parameters modeled, and Box-Cox Cole Green (BCCGo) distribution], LMST [an extension of LMS with 4 parameters being modeled and Box-Cox *t* distribution (BCTo)], and LMSP [also an extension of LMS to model 4 parameters, but using Box-Cox Power Exponential distribution (BCPEo)]. This function does not require specifications of degrees of freedom of parameters or smoothers for each of them (24). The software chooses the best specifications of the models based on the generalized Akaike's information criteria. The LMS function is recommended when the interest is on modeling growth charts and extracting percentiles (25). Given that the distributions used in the LMS function do not work with negative values, a constant of 20 kg was added to all GWG measures before fitting the models.

To evaluate overfitting, the degrees of freedom of each parameter in each model were examined, to ensure high values (>10) were not used in the model adjustment by the LMS function. We conducted the following model diagnostics: graph of the fitted parameters against gestational age; graph of the residuals from the models against fitted values of μ, kernel density estimate, and normal Quantile-Quantile (QQ) plots; and summary of quantile residuals and Q-stats (26). The worm plots created by van Buuren and Fredriks (27) were also examined. More importantly, at the end of the process, the estimates of the percentages of measurements below and above some selected percentiles (3^rd^, 10^th^, 25^th^, 50^th^, 75^th^, 90^th^, and 97^th^) were generated (internal validation of the model) (28).

We used the models’ estimates to produce z-scores, and percentiles were extracted according to gestational age, based on the prediction of the 4 parameters and the type of distribution (BCTo or BCPEo). We used the function “y2z” from the AGD package (29). To create the graphs and extract the values of the z-scores and percentiles, the 20 kg constant was subtracted from all GWG values.

### External validation

To assess the external validity of the charts, data from pregnant women collected in the Birth in Brazil study (2011) were used. Birth in Brazil was a nationwide and representative hospital-based study conducted between February 2011 and July 2012 in all 27 Brazilian federative units. The main goal of Birth in Brazil was to investigate the national incidence of cesarean sections, as well as its predictors and consequences. The study is described in detail elsewhere (30).

The initial sample size of Birth in Brazil was 23,955 women. For 15,115 women, body weight data from the pregnant booklets were photographed, then entered into a spreadsheet and revised for quality control purposes. We performed data cleaning using similar procedures to the ones implemented for the BMCNC data set (21). For the external validation, we applied the same eligibility criteria used in the construction of the charts. After applying the exclusion criteria, 4711 women with 31,052 weight measurements were available for analysis (**Supplementary Figure 1**).

**FIGURE 1:**
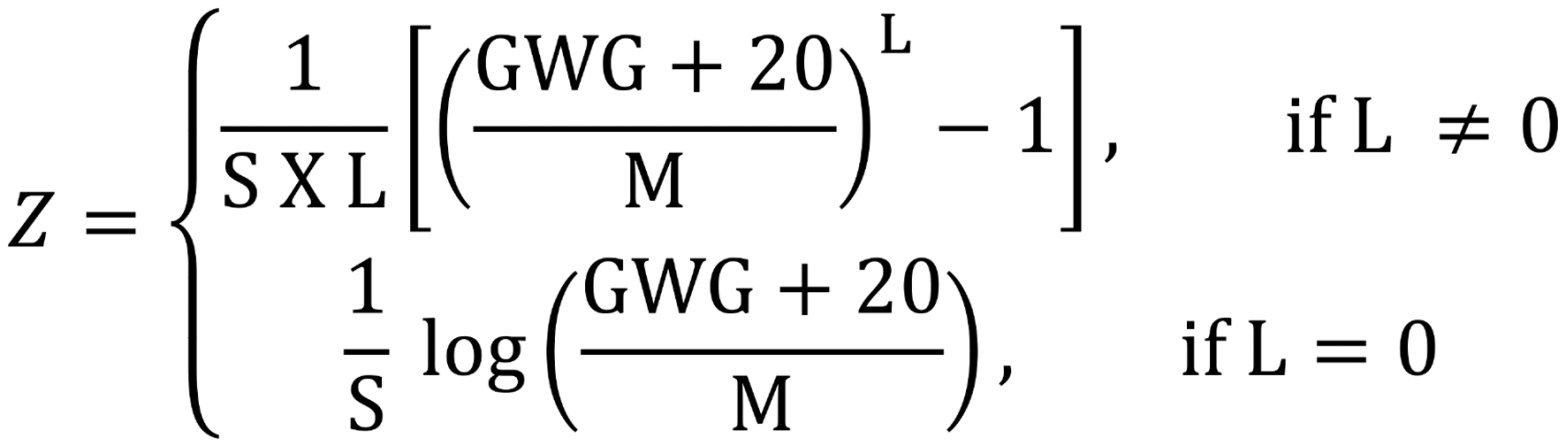
Equation to extract z-scores from GAMLSS models (Box-Cox *t* and Box-Cox Power Exponential distributions). The constant added to all models was 20 kg. Abbreviations: GAMLSS, Generalized Additive Models for Location, Scale, and Shape; GWG, gestational weight gain; L, lambda (or nu), M, mu; S, sigma.

We converted each weight measurement into a gestational age– and pre-pregnancy BMI–specific z-score (and percentile) using our new charts (**Figure 1**). Then, the percentages of women above/below the selected percentiles (50^th^; 25^th^/75^th^; 10^th^/90^th^; 3^rd^/97^th^) were determined. We expected that if the models were not overfitted to the BMCNC data, the percentages above/below those values in the Birth in Brazil study would be similar to the expected values: for example, 25% of the observations would be below the 25^th^ percentile and 25% would be above the 75^th^ percentile. This would also support the application of our charts to the general Brazilian population of pregnant women. All the analyses were conducted in Stata version 15 (StataCorp) and R, version 3.6 (R Foundation for Statistical Computing), and all the codes are available in Supplementary Methods 1.

## Results

**FIGURE 2:**
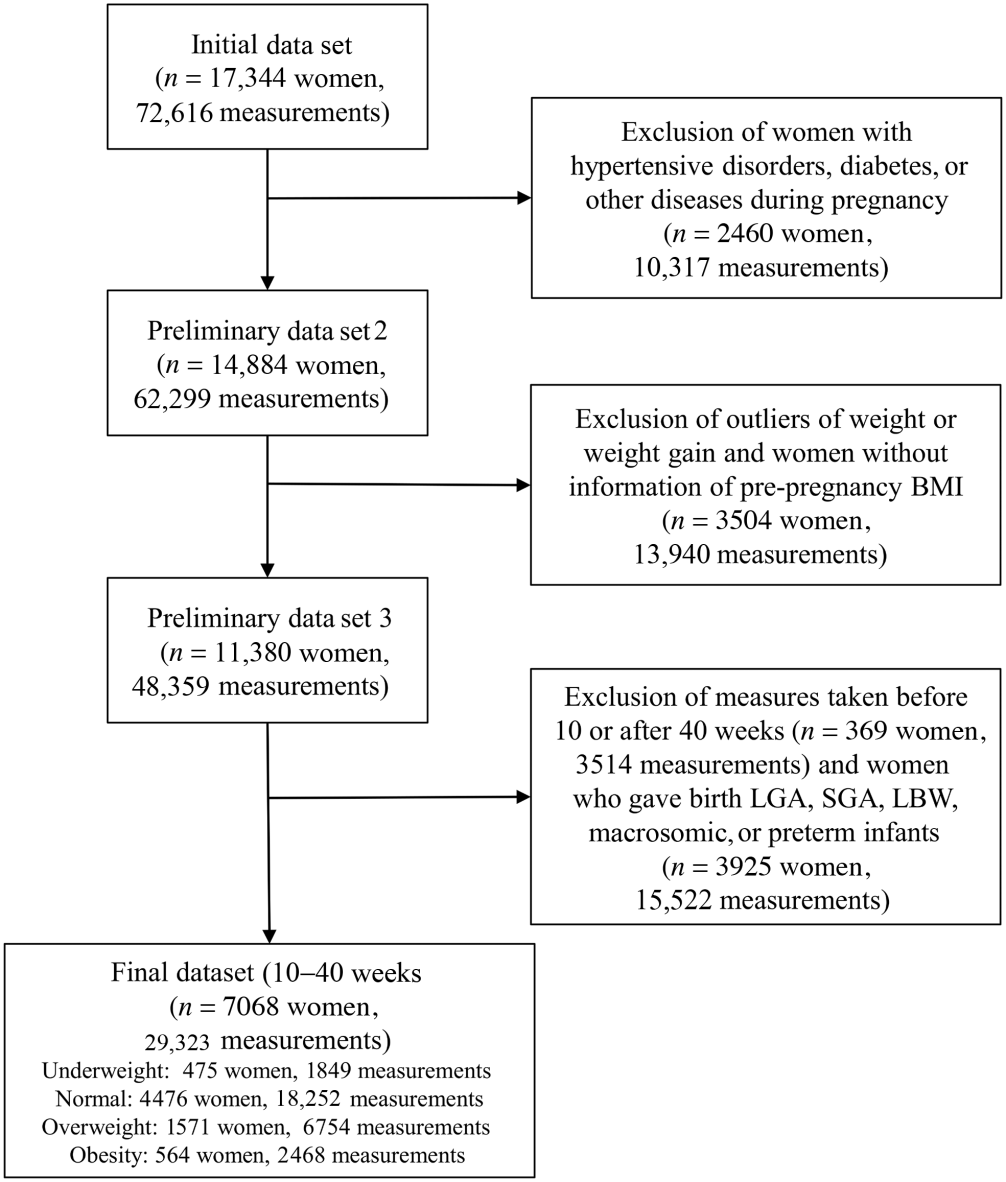
Sample size for the construction of the charts. “Measurements” refer to information of both gestational age and weight gain. Underweight, BMI < 18.5; normal weight, BMI ≥ 18.5 and < 25.0; overweight, BMI ≥ 25.0 and < 30.0; and obesity, BMI ≥ 30.0. Abbreviations: LBW, low birth weight; LGA, large for gestational age; SGA, small for gestational age.

**FIGURE 3:**
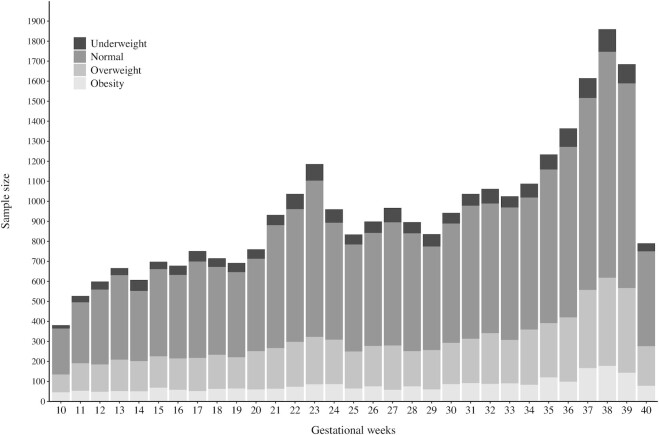
Sample size according to gestational age for each maternal pre-pregnancy BMI group (underweight, BMI < 18.5; normal weight, BMI ≥ 18.5 and < 25.0; overweight, BMI ≥ 25.0 and < 30.0; and obesity, BMI ≥ 30.0).

We used a cleaned data set of the BMCNC, comprising 17,344 women and 72,616 weight measurements. After applying the exclusion criteria, the final cohort included 7086 women with 29,323 weight gain measurements (median, 3; IQR, 2–5 measurements per woman; **Figure 2**). The highest proportion of women in the cohort were normal weight, followed by those with overweight, obesity, and underweight, respectively (**Figure 3**).

The median age of women in the cohort was 26 y (IQR, 22–31 y). The median age varied according to pre-pregnancy BMI, from 23 y in women with underweight to 27 y in women with obesity. Although the median birth weight was statistically different across the BMI categories (*P* < 0.001), the medians and IQRs of birth weight and length were remarkably similar across the categories. Women with obesity were more likely to have a cesarean delivery (54.3%) than women in other BMI categories. Most women (47.7%) had 9–11 y of education, lived with a partner (85.6%), and were multiparous (62.8%). Although significant differences were observed across the categories of pre-pregnancy BMI, differences in other demographic and neonatal characteristics were relatively small (**Table 1**).

**TABLE 1:** Maternal and neonatal characteristics of pregnancies from the Brazilian Maternal and Child Nutrition Consortium included in the creation of the Brazilian gestational weight gain charts (*n* = 7086 women)

	Nutritional status					
Maternal and neonatal characteristics	All women	Underweight: BMI <18.5	Normal weight: BMI ≥18.5 and <25.0	Overweight: BMI ≥25.0 and <30.0	Obesity: BMI ≥30.0	*P* value
Continuous variables, *n*, median (IQR)						
Maternal age, years	7085	475	4476	1570	564	<0.001*
	26 (22–31)	23 (21–27)	26 (22–30)	28 (23–32)	27 (23–32)	
Number of prenatal visits	29,323	1849	18,252	6754	2468	<0.001*
	3 (2–5)	3 (2–5)	3 (2–5)	3 (2–6)	4 (2–6)	
Birth weight, g	7086	475	4476	1571	564	<0.001*
	3250 (3030–3460)	3115 (2950–3340)	3240 (3025–3450)	3295 (3070–3500)	3300 (3080–3500)	
Length at birth, cm	6036	402	3830	1327	477	0.093*
	49 (48–50)	49 (48–50)	49 (48–50)	49 (48–50)	49 (48–50)	
Categorical variables, *n* (%)						
Maternal education, years	7068	474	4465	1567	562	0.013**
0–4	828 (11.7)	56 (11.8)	521 (11.7)	187 (11.9)	64 (11.4)	
5–8	1873 (26.5)	123 (26.0)	1181 (26.5)	418 (26.7)	151 (26.9)	
9–11	3368 (47.7)	259 (54.6)	2114 (47.3)	732 (46.7)	263 (46.8)	
12 or more	999 (14.1)	36 (7.6)	649 (14.5)	230 (14.7)	84 (14.9)	
Marital status	6028	425	3897	1291	415	0.034**
Does not live with a partner	871 (14.4)	76 (17.9)	578 (14.8)	163 (12.6)	54 (13.0)	
Lives with a partner	5157 (85.6)	349 (82.1)	3319 (85.2)	1128 (87.4)	361 (87.0)	
Previous pregnancies	6643	435	4220	1469	519	<0.001**
First pregnancy	2471 (37.2)	198 (45.5)	1643 (38.9)	488 (33.2)	142 (27.4)	
Second pregnancy or more	4172 (62.8)	237 (54.5)	2577 (61.1)	981 (66.8)	377 (72.6)	
Mode of delivery	6962	465	4389	1550	558	<0.001**
Normal	3986 (57.3)	314 (67.5)	2598 (59.2)	819 (52.8)	255 (45.7)	
Cesarean	2976 (42.7)	151 (32.5)	1791 (40.8)	731 (47.2)	303 (54.3)	

Variation in the sample sizes is due to missing data.

*P value for the Kruskal-Wallis test.

**P value for the chi-squared test.

The final models for under- and normal-weight women used a BCTo distribution, and the models for women with overweight and obesity used a BCPEo distribution. The final models for the charts using GAMLSS showed no evidence of overfitting (degrees of freedom for all parameters were <10; **Supplementary Table 1**). All the diagnostic procedures had satisfactory results, with occasional minor inadequacies (data available on request).

**FIGURE 4:**
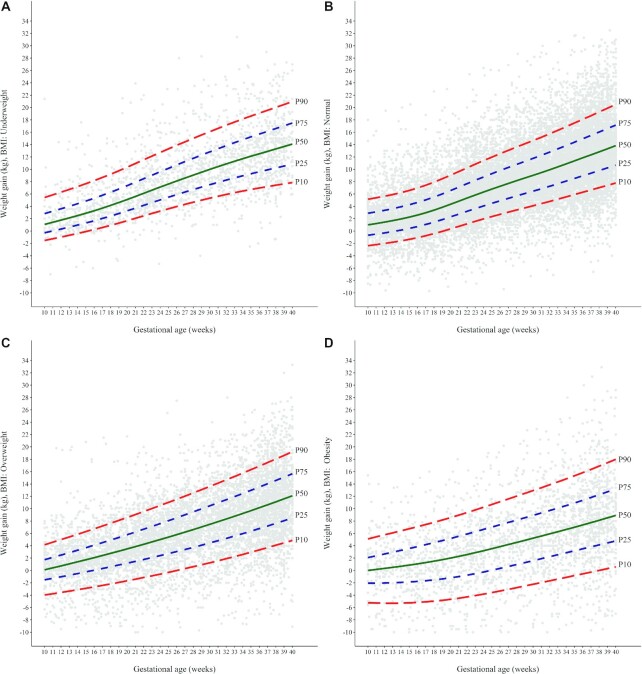
Gestational weight gain charts according to pre-pregnancy BMI for Brazilian women, using data from the Brazilian Maternal and Child Nutrition Consortium. The gray dots represent each weight gain measurement used in the construction of the charts. (A) Underweight (BMI < 18.5; *n* = 1849 measurements); (B) normal weight (BMI ≥ 18.5 and < 25.0; *n* = 18,252 measurements); (C) overweight (BMI ≥ 25.0 and < 30.0; *n* = 6754 measurements); and (D) obesity (BMI ≥ 30.0; *n* = 2468 measurements).

**Figure 4** shows selected percentiles of GWG according to gestational age and pre-pregnancy BMI. The GWG distribution varied substantially when different BMI categories were compared. Women with under-, normal-, and overweight had relatively similar distributions, while those with obesity had a considerably different GWG pattern, with systematically lower centiles than those estimated for the other categories. The median amounts of GWG at 40 weeks were 14.1 kg (IQR, 10.8–17.5 kg) for women with underweight; 13.8 kg (IQR, 10.7–17.2 kg) for normal-weight women; 12.1 kg (IQR, 8.5–15.7 kg) for women with overweight; and 8.9 kg (IQR, 4.8–13.2 kg) for women with obesity (**Supplementary Tables 2**–**5**).

Our internal validation confirmed that an appropriate proportion of measurements fell below the corresponding percentile on the chart for all BMI categories. For example, 3.1% of normal-weight women were below the 3^rd^ percentile, 9.6% below the 10^th^, 89.4% below the 90^th^, and 96.6% below the 97^th^ (**Table 2**). All variation from the expected percentages was <1%. Results for the external validation showed that the percentages were close to those expected, with differences varying from 0.1–3.4%. The largest differences between the expected and observed values were in women with pre-pregnancy overweight and obesity, specifically in the 25^th^, 50^th^, and 75^th^ percentiles. For these categories, the differences were smaller in the extreme selected percentiles (3^rd^, 10^th^, 90^th^, and 97^th^).

**TABLE 2:** Performance of the gestational weight gain charts in the internal and external validation procedures

	% of observations below/above the selected centiles; internal validation, BMCNC data							% of observations below/above the selected centiles; external validation, Birth in Brazil data						
BMI category, kg/m^2^	<P3	<P10	<P25	<P50	<P75	<P90	<P97	<P3	<P10	<P25	<P50	<P75	<P90	<P97
Underweight: <18.5	2.7	9.0	24.9	50.9	75.6	89.0	96.7	2.6	8.2	22.4	49.6	73.2	88.8	96.5
Normal weight: ≥18.5 and <25.0	3.1	9.6	24.4	50.9	75.0	89.4	96.6	2.4	8.2	23.0	51.6	75.7	89.5	96.5
Overweight: ≥25.0 and <30.0	3.1	9.9	24.3	49.9	74.7	89.9	97.1	2.0	7.6	21.6	46.8	71.7	87.8	95.5
Obesity: ≥30.0	3.6	10.6	24.8	74.3	50.4	89.9	96.6	3.9	10.1	23.2	53.0	77.5	90.7	96.6

Abbreviation: BMCNC, Brazilian Maternal and Child Nutrition Consortium.

## Discussion

In this study, prescriptive GWG charts were constructed for each pre-pregnancy BMI category, according to WHO cutoffs, using appropriate statistical models with no overfitting and with good results in the internal and external validation procedure. These charts describe patterns of GWG among Brazilian women with good pregnancy outcomes and provide an important tool for monitoring weight gain in pregnancy in clinical care, especially in Latin America and other LMICs. In **Supplementary Methods 2**, we provide a worked example of how to convert a woman's weight gain measurement into a gestational age– and BMI-specific weight z-score and percentile.

The pattern of GWG varied substantially according to pre-pregnancy BMI and highlighted the nonlinearity of weight gain during pregnancy. For women classified as underweight, normal weight, or overweight, a small amount of weight gain was observed during the first trimester (median 2.1 kg for underweight, 1.7 kg for normal weight, and 1.1 kg for overweight). This pattern was different for women with obesity, who had lower first-trimester weight gain values and even weight loss in this period (median, 0.5 kg; IQR, −2.0 to 3.0 kg). The pattern of GWG for the second and third trimesters showed a higher rate of gain as compared with the first trimester, and the differences among the BMI categories became more evident. The median GWG at the 40^th^ week varied between 9 and 14 kg among women with obesity and underweight, respectively. Many women with obesity experienced weight loss at the end of pregnancy, a worrying observation because weight loss during pregnancy is not recommended (15, 31).

The internal validation of the model produced particularly good results, ensuring that the percentiles plotted in the charts accurately represent the distribution of GWG according to gestational age and pre-pregnancy BMI among Brazilian women, without any indication of bias. External validation results were also satisfying, ensuring that the models were not overfitted to the data and that the distribution of GWG depicted in our charts is similar to the distribution of GWG in another large sample of Brazilian women.

Although GWG is commonly monitored as part of the prenatal care routine in several countries (32), the WHO does not provide any guidance regarding appropriate GWG in its antenatal care manual (33). Given the lack of an international reference on GWG, several charts have been created in recent years. Santos et al. (9) developed GWG charts for women from Europe, North America, and Oceania using data pooled from studies conducted in multiple countries. The patterns of weight gain observed among women with overweight and obesity throughout pregnancy and among women from all BMI categories during the second and third trimesters in Santos et al. (9) differed from the patterns observed in the current study. Their charts described a small reduction in GWG among women with overweight between 20–25 weeks. Moreover, there are weeks during the second and third trimesters where the curves are flat (no weight gain seems to occur) for women with obesity. These characteristics of the charts by Santos et al. (9) may have resulted from their choice of the internal breakpoints for μ in the GAMLSS models that they constructed, which may have led to an artificial distribution of GWG, especially among women with overweight and obesity. Also, the data set used by Santos et al. (9) had relatively few measurements per woman, so it could be that they were picking up a cross-sectional effect of different women at different ages, rather than true decreases or leveling of curves. Using the LMS function in the current study, no breakpoints were chosen for any of the parameters modeled. In addition, our study relies on measured data on weight during pregnancy, while some studies incorporated by Santos et al. (9) only included self-reported weight.

Another important chart was developed by INTERGROWTH-21^st^, a multicenter and multiethnic study conducted in 8 countries, including Brazil (8). The pattern of weight gain in the current study is similar to that in the INTERGROWTH-21^st^ GWG chart for normal-weight women, even though the INTERGROWTH-21^st^ chart used weight measured between 9–14 weeks and not a self-reported pre-pregnancy weight to calculate GWG. INTERGROWTH-21^st^ was able to produce charts only for normal-weight women and not for women in other BMI categories.

The patterns of GWG observed in those charts created in the United States and China (7, 10) also differ from what we observed for Brazilian women, especially regarding the rate of GWG. It is important to mention, however, that the methods used by these groups differ from the methods we employed. The US charts are based on a population that includes a substantial number of women with overweight or obesity, many of whom experienced weight loss during pregnancy. These facts may help to explain the differences in the GWG trajectories observed between their charts and ours. Also, the US study was based on medical records from a single hospital in Pittsburgh, with mostly non-Hispanic White women. The Chinese charts used different BMI cutoffs and restricted cubic spline models with an elevated number of knots (*n* = 8). This may explain the difference in the GWG patterns between the Chinese and Brazilian charts.

The values for the medians and IQRs for total GWG at the 40^th^ week from the current study were also compared to the IOM GWG recommendations (15), currently in use in Brazil and many other countries (32). The ranges proposed by the IOM corresponded to approximately the 25^th^ to 75^th^ percentiles of our new charts for under- and normal-weight women (underweight: Brazil IQR 10.8–17.5 kg vs. IOM 12.5–18 kg; normal weight: Brazil IQR 10.7–17.2 kg vs. IOM 11.5–16 kg). In contrast, the upper limit of the IOM recommendation was markedly lower than the 75^th^ percentile on our charts (Brazil: 15.7 vs. IOM 11.5 kg, in women with overweight and Brazil 13.2 kg vs. IOM 9 kg, in women with obesity). For these BMI categories, the upper limits of the IOM recommendations corresponded to approximately the 45^th^ and 50^th^ percentiles of our chart, respectively. Nevertheless, the median and IQR values are not GWG recommendations, which are yet to be developed for Brazilian women using appropriate methods to evaluate the relationships between these GWG ranges and maternal and perinatal outcomes.

The development of the BMCNC is a pioneer initiative in Brazil that combined 21 Brazilian cohort studies to provide harmonized and homogeneous data for this study. The use of this data set yielded a substantial number of weight and gestational age measurements to construct GWG charts according to BMI categories. The use of pre-pregnancy weight (rather than early pregnancy weight) to calculate the GWG and BMI enabled us to create charts starting at the 10^th^ gestational week, which permits an evaluation of weight gain at least for the last month of the first trimester of pregnancy. This is important, as it allows the monitoring to start early enough in pregnancy that interventions and counseling (if developed and delivered) could help prevent excessive GWG and the adverse outcomes related to it. In addition, previous work by our team (19) showed that self-reported pre-pregnancy weight is easily obtained and commonly available in public health-care services in Brazil. As a result, having this information available for the calculation of GWG would coordinate well with the procedures used in routine prenatal care.

The evaluation of several statistical models to create the charts and the adoption of the best model after considering several measurements of diagnostics and internal and external validations are other strengths of this study. A characteristic that differentiates our study from others in the field is our use of an external validation procedure, which was based on a national data set collected in the process of routine prenatal care.

Despite the important strengths, our study also has some limitations. Unfortunately, we did not have sufficient data to separate obesity into 3 classes, as in the WHO cutoffs (23). This resulted from the low proportion of women in obesity classes II and III in Brazil (approximately 3.3% and 1%, respectively; data from the Food and Nutrition Surveillance System 2008–2018, not shown). We believe that not having separate charts for those categories does not represent a constraint to the use of these charts in the public health-care system. We also did not have enough data before the 10^th^ gestational week to include even earlier weeks in the charts. In Brazil, where many women start prenatal care after the first trimester (20), this would represent a minor constraint.

The evidence derived from the current study will result in major changes in the nutritional assessment of pregnant women in Brazil, shifting the tool that monitors GWG from BMI to the cumulative GWG. GWG charts represent a useful monitoring tool in prenatal care services because they are simple, easy to calculate and to understand. Having a tool that allows women to continuously monitor their GWG can increase awareness of this important measure, which should not be neglected, and can help health-care practitioners in interventions to ensure adequate GWG. Those interventions can begin early in pregnancy, since the proposed charts start during the first trimester.

## Supplementary Material

nqaa402_Supplemental_Files

## Data Availability

The Brazilian Maternal and Child Nutrition Consortium is managed by a team of researchers from the Nutritional Epidemiology Observatory, from the Nutrition Institute, in the Federal University of Rio de Janeiro. Data sets are not yet available for public use, but requests can be made to the coordinator of the project (gilberto.kac@gmail.com) and the whole consortium group is consulted regarding data sharing for specific studies. The codes used for the analyses are available in Supplementary Methods 1.

## Notes

This work was supported by the Brazilian National Research Council (CNPq) and the Brazilian Ministry of Health (grant numbers 408678/2017-8 and 443770/2018-2) and Bill and Melinda Gates Foundation (grant number OPP 1202165). Birth in Brazil received financial support from the CNPq and the National School of Public Health, Oswaldo Cruz Foundation, Brazil.

The funders did not play any role in the design, implementation, analyses, and interpretation of the data.

Supplemental Tables 1–5 and Supplementary Methods 1 and 2 are available from the “Supplementary data” link in the online posting of the article and from the same link in the online table of contents at https://academic.oup.com/ajcn/.

Abbreviations used: BCPEo, Box-Cox power exponential distribution; BCTo, Box-Cox *t* distribution; BMCNC, Brazilian Maternal and Child Nutrition Consortium; GAMLSS, Generalized Additive Models for Location, Scale, and Shape; GWG, Gestational weight gain; INTERGROWTH-21^st^, International Fetal and Newborn Growth Consortium for the 21^st^ Century; IOM, Institute of Medicine; LGA, large for gestational age; LMIC, low- and middle-income country; LMS, lambda, mu, sigma; SGA, small for gestational age.
