# Supplementary material for: Gestational weight gain charts: results from the Brazilian Maternal and Child Nutrition Consortium
Source: Am J Clin Nutr. 2021 Mar 19;113(5):1351–60. doi: 10.1093/ajcn/nqaa402 (PMC8106749; doi:10.1093/ajcn/nqaa402)
Supplement: nqaa402_Supplemental_Files [file nqaa402_supplemental_files.zip › Sup_tables_AJCN_27-10-20.docx]

**ONLINE SUPPLEMENTARY MATERIAL**

**Gestational weight gain charts: Results from the Brazilian Maternal and Child Nutrition Consortium**

First authors: Gilberto Kac, Thais R. B. Carrilho

**SUPPLEMENTARY TABLES**

Supplementary table 1. Specifications of the Generalized Additive model for location, scale, and shape (GAMLSS) adjusted to each BMI category

|  | **Specifications of the model** | | | | |
| --- | --- | --- | --- | --- | --- |
| **BMI category** | Distribution | DF (mu) | DF (sigma) | DF (nu) | DF (tau) |
| Underweight  (n=1849 observations) | BCTo | 4.22 | 2.00 | 2.00 | 2.00 |
| Normal weight (n=18252 observations) | BCTo | 6.80 | 2.04 | 2.00 | 2.00 |
| Overweight  (n= 6754 observations) | BCPEo | 2.41 | 2.01 | 2.00 | 3.33 |
| Obesity  (n=2468 observations) | BCPEo | 2.80 | 2.00 | 2.00 | 4.00 |

BCTo: Box-Cox *t* with log as the link function for µ; BCPEo: Box-Cox Power Exponential with log as the link function for µ; DF: degrees of freedom; BMI cutoffs: Underweight, BMI < 18.5 kg/m^2^; Normal, BMI ≥ 18.5 and < 25.0 kg/m^2^; Overweight, BMI ≥ 25.0 and < 30.0 kg/m^2^ and Obesity, BMI ≥ 30.0 kg/m^2^.

Supplementary table 2. Week-specific Box-Cox *t* model parameters and selected percentiles of gestational weight gain for pre-pregnancy underweight, Brazilian Maternal and Child Nutrition Consortium data.

| Gestational age (weeks) | Model parameters | | | | Percentiles of gestational weight gain (kg) | | | | | | | | | | | | | | | |
| --- | --- | --- | --- | --- | --- | --- | --- | --- | --- | --- | --- | --- | --- | --- | --- | --- | --- | --- | --- | --- |
|  | mu | sigma | nu | tau | P1 | P2.3 | P3 | P5 | P10 | P16 | P20 | P25 | P50 | P75 | P80 | P84 | P90 | P95 | P97.7 | P99 |
| 10 | 21.126 | 0.097 | -2.582 | 3.026 | -4.3 | -3.3 | -3.0 | -2.4 | -1.5 | -0.9 | -0.6 | -0.3 | 1.1 | 2.8 | 3.4 | 4.0 | 5.4 | 8.2 | 12.7 | 20.3 |
| 11 | 21.464 | 0.098 | -2.479 | 3.139 | -4.0 | -3.0 | -2.7 | -2.1 | -1.2 | -0.6 | -0.3 | 0.0 | 1.4 | 3.2 | 3.8 | 4.4 | 5.9 | 8.6 | 13.2 | 20.8 |
| 12 | 21.809 | 0.099 | -2.377 | 3.255 | -3.8 | -2.8 | -2.4 | -1.8 | -1.0 | -0.3 | 0.0 | 0.3 | 1.8 | 3.6 | 4.2 | 4.8 | 6.3 | 9.1 | 13.7 | 21.4 |
| 13 | 22.161 | 0.101 | -2.274 | 3.376 | -3.5 | -2.5 | -2.2 | -1.6 | -0.7 | 0.0 | 0.3 | 0.6 | 2.1 | 4.0 | 4.6 | 5.3 | 6.8 | 9.6 | 14.1 | 21.9 |
| 14 | 22.526 | 0.102 | -2.172 | 3.502 | -3.2 | -2.2 | -1.9 | -1.3 | -0.4 | 0.3 | 0.6 | 1.0 | 2.5 | 4.4 | 5.1 | 5.7 | 7.2 | 10.0 | 14.6 | 22.3 |
| 15 | 22.907 | 0.103 | -2.069 | 3.632 | -3.0 | -2.0 | -1.6 | -1.0 | -0.1 | 0.6 | 0.9 | 1.3 | 2.9 | 4.9 | 5.5 | 6.2 | 7.7 | 10.5 | 15.0 | 22.8 |
| 16 | 23.306 | 0.104 | -1.967 | 3.767 | -2.7 | -1.7 | -1.3 | -0.7 | 0.2 | 0.9 | 1.3 | 1.7 | 3.3 | 5.3 | 6.0 | 6.6 | 8.2 | 11.0 | 15.5 | 23.1 |
| 17 | 23.727 | 0.106 | -1.864 | 3.908 | -2.4 | -1.4 | -1.0 | -0.4 | 0.6 | 1.3 | 1.7 | 2.1 | 3.7 | 5.8 | 6.5 | 7.1 | 8.7 | 11.5 | 15.9 | 23.4 |
| 18 | 24.170 | 0.107 | -1.762 | 4.053 | -2.1 | -1.1 | -0.7 | 0.0 | 0.9 | 1.7 | 2.0 | 2.4 | 4.2 | 6.3 | 7.0 | 7.6 | 9.2 | 12.0 | 16.4 | 23.7 |
| 19 | 24.635 | 0.108 | -1.660 | 4.204 | -1.8 | -0.7 | -0.4 | 0.3 | 1.3 | 2.0 | 2.4 | 2.9 | 4.6 | 6.8 | 7.5 | 8.2 | 9.8 | 12.6 | 16.9 | 23.9 |
| 20 | 25.118 | 0.109 | -1.557 | 4.360 | -1.5 | -0.4 | 0.0 | 0.7 | 1.7 | 2.5 | 2.9 | 3.3 | 5.1 | 7.4 | 8.1 | 8.8 | 10.4 | 13.1 | 17.3 | 24.1 |
| 21 | 25.614 | 0.111 | -1.455 | 4.522 | -1.2 | -0.1 | 0.3 | 1.0 | 2.1 | 2.9 | 3.3 | 3.7 | 5.6 | 7.9 | 8.6 | 9.3 | 10.9 | 13.7 | 17.8 | 24.4 |
| 22 | 26.118 | 0.112 | -1.352 | 4.690 | -0.9 | 0.3 | 0.6 | 1.4 | 2.5 | 3.3 | 3.7 | 4.2 | 6.1 | 8.5 | 9.2 | 9.9 | 11.5 | 14.3 | 18.3 | 24.5 |
| 23 | 26.624 | 0.113 | -1.250 | 4.865 | -0.6 | 0.6 | 1.0 | 1.8 | 2.9 | 3.7 | 4.1 | 4.6 | 6.6 | 9.0 | 9.8 | 10.5 | 12.1 | 14.9 | 18.8 | 24.7 |
| 24 | 27.126 | 0.115 | -1.147 | 5.046 | -0.3 | 0.9 | 1.3 | 2.1 | 3.2 | 4.1 | 4.6 | 5.1 | 7.1 | 9.6 | 10.4 | 11.1 | 12.7 | 15.5 | 19.2 | 24.8 |
| 25 | 27.622 | 0.116 | -1.045 | 5.234 | 0.0 | 1.2 | 1.6 | 2.4 | 3.6 | 4.5 | 5.0 | 5.5 | 7.6 | 10.2 | 10.9 | 11.7 | 13.3 | 16.0 | 19.7 | 25.0 |
| 26 | 28.109 | 0.118 | -0.942 | 5.428 | 0.3 | 1.5 | 2.0 | 2.8 | 4.0 | 4.9 | 5.4 | 5.9 | 8.1 | 10.7 | 11.5 | 12.2 | 13.9 | 16.6 | 20.1 | 25.1 |
| 27 | 28.589 | 0.119 | -0.840 | 5.630 | 0.5 | 1.8 | 2.2 | 3.1 | 4.3 | 5.3 | 5.8 | 6.3 | 8.6 | 11.3 | 12.0 | 12.8 | 14.5 | 17.1 | 20.6 | 25.3 |
| 28 | 29.062 | 0.121 | -0.738 | 5.840 | 0.8 | 2.1 | 2.5 | 3.4 | 4.7 | 5.7 | 6.2 | 6.7 | 9.1 | 11.8 | 12.6 | 13.4 | 15.0 | 17.6 | 21.0 | 25.5 |
| 29 | 29.529 | 0.122 | -0.635 | 6.057 | 1.0 | 2.3 | 2.8 | 3.7 | 5.0 | 6.0 | 6.5 | 7.1 | 9.5 | 12.3 | 13.1 | 13.9 | 15.6 | 18.2 | 21.5 | 25.7 |
| 30 | 29.988 | 0.123 | -0.533 | 6.282 | 1.2 | 2.6 | 3.1 | 4.0 | 5.3 | 6.4 | 6.9 | 7.5 | 10.0 | 12.8 | 13.6 | 14.4 | 16.1 | 18.7 | 21.9 | 26.0 |
| 31 | 30.438 | 0.125 | -0.430 | 6.516 | 1.4 | 2.8 | 3.3 | 4.2 | 5.6 | 6.7 | 7.3 | 7.9 | 10.4 | 13.3 | 14.2 | 15.0 | 16.6 | 19.2 | 22.3 | 26.2 |
| 32 | 30.878 | 0.126 | -0.328 | 6.758 | 1.5 | 3.0 | 3.5 | 4.5 | 5.9 | 7.0 | 7.6 | 8.3 | 10.9 | 13.8 | 14.7 | 15.5 | 17.2 | 19.7 | 22.7 | 26.5 |
| 33 | 31.307 | 0.128 | -0.225 | 7.010 | 1.7 | 3.2 | 3.7 | 4.7 | 6.2 | 7.4 | 8.0 | 8.6 | 11.3 | 14.3 | 15.2 | 16.0 | 17.7 | 20.2 | 23.2 | 26.7 |
| 34 | 31.724 | 0.129 | -0.123 | 7.271 | 1.8 | 3.4 | 3.9 | 5.0 | 6.5 | 7.7 | 8.3 | 9.0 | 11.7 | 14.8 | 15.6 | 16.5 | 18.2 | 20.6 | 23.6 | 27.0 |
| 35 | 32.129 | 0.131 | -0.020 | 7.541 | 1.9 | 3.5 | 4.1 | 5.2 | 6.7 | 8.0 | 8.6 | 9.3 | 12.1 | 15.3 | 16.1 | 16.9 | 18.6 | 21.1 | 24.0 | 27.3 |
| 36 | 32.524 | 0.133 | 0.082 | 7.822 | 2.0 | 3.7 | 4.2 | 5.3 | 7.0 | 8.2 | 8.9 | 9.6 | 12.5 | 15.7 | 16.6 | 17.4 | 19.1 | 21.6 | 24.4 | 27.6 |
| 37 | 32.913 | 0.134 | 0.185 | 8.113 | 2.0 | 3.8 | 4.4 | 5.5 | 7.2 | 8.5 | 9.2 | 9.9 | 12.9 | 16.2 | 17.0 | 17.9 | 19.6 | 22.0 | 24.7 | 27.9 |
| 38 | 33.302 | 0.136 | 0.287 | 8.414 | 2.1 | 3.9 | 4.5 | 5.7 | 7.4 | 8.8 | 9.5 | 10.2 | 13.3 | 16.6 | 17.5 | 18.3 | 20.0 | 22.4 | 25.1 | 28.1 |
| 39 | 33.692 | 0.137 | 0.389 | 8.727 | 2.1 | 4.0 | 4.6 | 5.8 | 7.6 | 9.0 | 9.8 | 10.5 | 13.7 | 17.1 | 17.9 | 18.8 | 20.5 | 22.9 | 25.5 | 28.5 |
| 40 | 34.085 | 0.139 | 0.492 | 9.052 | 2.1 | 4.0 | 4.7 | 6.0 | 7.9 | 9.3 | 10.0 | 10.8 | 14.1 | 17.5 | 18.4 | 19.3 | 21.0 | 23.3 | 25.9 | 28.8 |

Supplementary table 3. Week-specific Box-Cox *t* model parameters and selected percentiles of gestational weight gain for pre-pregnancy normal weight, Brazilian Maternal and Child Nutrition Consortium data.

| Gestational age (weeks) | Model parameters | | | | Percentiles of gestational weight gain (kg) | | | | | | | | | | | | | | | |
| --- | --- | --- | --- | --- | --- | --- | --- | --- | --- | --- | --- | --- | --- | --- | --- | --- | --- | --- | --- | --- |
|  | mu | sigma | nu | tau | P1 | P2.3 | P3 | P5 | P10 | P16 | P20 | P25 | P50 | P75 | P80 | P84 | P90 | P95 | P97.7 | P99 |
| 10 | 21.012 | 0.113 | -0.147 | 3.590 | -6.4 | -4.9 | -4.4 | -3.6 | -2.4 | -1.5 | -1.1 | -0.7 | 1.0 | 2.9 | 3.4 | 4.0 | 5.2 | 7.1 | 9.8 | 13.5 |
| 11 | 21.224 | 0.114 | -0.126 | 3.719 | -6.2 | -4.7 | -4.3 | -3.4 | -2.2 | -1.4 | -0.9 | -0.5 | 1.2 | 3.1 | 3.7 | 4.2 | 5.4 | 7.3 | 9.9 | 13.5 |
| 12 | 21.448 | 0.115 | -0.106 | 3.854 | -6.0 | -4.5 | -4.1 | -3.2 | -2.0 | -1.2 | -0.7 | -0.3 | 1.5 | 3.4 | 3.9 | 4.5 | 5.6 | 7.6 | 10.1 | 13.6 |
| 13 | 21.690 | 0.115 | -0.085 | 3.993 | -5.8 | -4.3 | -3.9 | -3.0 | -1.8 | -1.0 | -0.5 | -0.1 | 1.7 | 3.6 | 4.2 | 4.7 | 5.9 | 7.8 | 10.3 | 13.7 |
| 14 | 21.958 | 0.116 | -0.064 | 4.137 | -5.6 | -4.1 | -3.7 | -2.8 | -1.6 | -0.7 | -0.3 | 0.2 | 2.0 | 3.9 | 4.5 | 5.1 | 6.2 | 8.1 | 10.6 | 13.9 |
| 15 | 22.257 | 0.117 | -0.044 | 4.286 | -5.4 | -3.9 | -3.4 | -2.6 | -1.3 | -0.5 | 0.0 | 0.4 | 2.3 | 4.3 | 4.8 | 5.4 | 6.6 | 8.5 | 10.9 | 14.1 |
| 16 | 22.593 | 0.118 | -0.023 | 4.441 | -5.1 | -3.6 | -3.2 | -2.3 | -1.1 | -0.2 | 0.3 | 0.7 | 2.6 | 4.6 | 5.2 | 5.8 | 7.0 | 8.9 | 11.2 | 14.4 |
| 17 | 22.972 | 0.118 | -0.002 | 4.602 | -4.8 | -3.3 | -2.9 | -2.0 | -0.8 | 0.1 | 0.6 | 1.1 | 3.0 | 5.1 | 5.6 | 6.2 | 7.4 | 9.3 | 11.7 | 14.7 |
| 18 | 23.394 | 0.119 | 0.019 | 4.768 | -4.5 | -3.0 | -2.6 | -1.7 | -0.4 | 0.5 | 0.9 | 1.4 | 3.4 | 5.5 | 6.1 | 6.7 | 7.9 | 9.8 | 12.2 | 15.2 |
| 19 | 23.853 | 0.120 | 0.040 | 4.940 | -4.2 | -2.7 | -2.2 | -1.3 | 0.0 | 0.9 | 1.4 | 1.9 | 3.9 | 6.0 | 6.6 | 7.2 | 8.5 | 10.4 | 12.7 | 15.7 |
| 20 | 24.342 | 0.121 | 0.060 | 5.119 | -3.8 | -2.3 | -1.8 | -0.9 | 0.4 | 1.3 | 1.8 | 2.3 | 4.3 | 6.6 | 7.2 | 7.8 | 9.0 | 11.0 | 13.3 | 16.2 |
| 21 | 24.844 | 0.122 | 0.081 | 5.303 | -3.4 | -1.9 | -1.5 | -0.5 | 0.8 | 1.7 | 2.2 | 2.8 | 4.8 | 7.1 | 7.8 | 8.4 | 9.6 | 11.6 | 13.9 | 16.8 |
| 22 | 25.347 | 0.122 | 0.102 | 5.495 | -3.1 | -1.6 | -1.1 | -0.1 | 1.2 | 2.2 | 2.7 | 3.2 | 5.4 | 7.7 | 8.3 | 9.0 | 10.2 | 12.2 | 14.5 | 17.4 |
| 23 | 25.844 | 0.123 | 0.122 | 5.694 | -2.7 | -1.2 | -0.7 | 0.2 | 1.6 | 2.6 | 3.1 | 3.6 | 5.8 | 8.2 | 8.9 | 9.5 | 10.8 | 12.8 | 15.1 | 18.0 |
| 24 | 26.330 | 0.124 | 0.143 | 5.899 | -2.4 | -0.9 | -0.4 | 0.6 | 2.0 | 3.0 | 3.5 | 4.1 | 6.3 | 8.8 | 9.4 | 10.1 | 11.4 | 13.4 | 15.7 | 18.5 |
| 25 | 26.803 | 0.125 | 0.164 | 6.112 | -2.1 | -0.5 | 0.0 | 0.9 | 2.4 | 3.4 | 3.9 | 4.5 | 6.8 | 9.3 | 10.0 | 10.6 | 12.0 | 14.0 | 16.3 | 19.1 |
| 26 | 27.263 | 0.126 | 0.185 | 6.333 | -1.8 | -0.2 | 0.3 | 1.3 | 2.7 | 3.8 | 4.3 | 4.9 | 7.3 | 9.8 | 10.5 | 11.2 | 12.5 | 14.5 | 16.9 | 19.6 |
| 27 | 27.714 | 0.126 | 0.205 | 6.562 | -1.5 | 0.1 | 0.6 | 1.6 | 3.1 | 4.2 | 4.7 | 5.3 | 7.7 | 10.3 | 11.0 | 11.7 | 13.1 | 15.1 | 17.4 | 20.2 |
| 28 | 28.159 | 0.127 | 0.226 | 6.799 | -1.2 | 0.4 | 0.9 | 2.0 | 3.4 | 4.5 | 5.1 | 5.7 | 8.2 | 10.8 | 11.5 | 12.2 | 13.6 | 15.6 | 18.0 | 20.7 |
| 29 | 28.597 | 0.128 | 0.247 | 7.045 | -0.9 | 0.7 | 1.3 | 2.3 | 3.8 | 4.9 | 5.5 | 6.1 | 8.6 | 11.3 | 12.0 | 12.7 | 14.1 | 16.2 | 18.5 | 21.2 |
| 30 | 29.032 | 0.129 | 0.268 | 7.299 | -0.6 | 1.0 | 1.6 | 2.6 | 4.1 | 5.2 | 5.8 | 6.5 | 9.0 | 11.8 | 12.5 | 13.2 | 14.7 | 16.7 | 19.1 | 21.8 |
| 31 | 29.473 | 0.130 | 0.288 | 7.563 | -0.3 | 1.3 | 1.9 | 2.9 | 4.5 | 5.6 | 6.2 | 6.9 | 9.5 | 12.3 | 13.0 | 13.7 | 15.2 | 17.3 | 19.6 | 22.3 |
| 32 | 29.931 | 0.131 | 0.309 | 7.836 | 0.0 | 1.6 | 2.2 | 3.2 | 4.8 | 6.0 | 6.6 | 7.3 | 9.9 | 12.8 | 13.6 | 14.3 | 15.8 | 17.9 | 20.2 | 22.9 |
| 33 | 30.404 | 0.131 | 0.330 | 8.119 | 0.3 | 2.0 | 2.5 | 3.6 | 5.2 | 6.4 | 7.0 | 7.7 | 10.4 | 13.3 | 14.1 | 14.8 | 16.3 | 18.4 | 20.8 | 23.5 |
| 34 | 30.885 | 0.132 | 0.350 | 8.413 | 0.6 | 2.3 | 2.8 | 3.9 | 5.5 | 6.8 | 7.4 | 8.1 | 10.9 | 13.9 | 14.6 | 15.4 | 16.9 | 19.0 | 21.4 | 24.1 |
| 35 | 31.371 | 0.133 | 0.371 | 8.717 | 0.9 | 2.6 | 3.2 | 4.3 | 5.9 | 7.2 | 7.8 | 8.5 | 11.4 | 14.4 | 15.2 | 16.0 | 17.5 | 19.7 | 22.0 | 24.7 |
| 36 | 31.857 | 0.134 | 0.392 | 9.032 | 1.2 | 2.9 | 3.5 | 4.6 | 6.3 | 7.6 | 8.2 | 9.0 | 11.9 | 14.9 | 15.8 | 16.5 | 18.1 | 20.3 | 22.7 | 25.3 |
| 37 | 32.344 | 0.135 | 0.413 | 9.358 | 1.5 | 3.2 | 3.8 | 5.0 | 6.7 | 8.0 | 8.6 | 9.4 | 12.3 | 15.5 | 16.3 | 17.1 | 18.7 | 20.9 | 23.3 | 25.9 |
| 38 | 32.833 | 0.136 | 0.433 | 9.696 | 1.8 | 3.5 | 4.1 | 5.3 | 7.0 | 8.4 | 9.1 | 9.8 | 12.8 | 16.0 | 16.9 | 17.7 | 19.3 | 21.5 | 23.9 | 26.6 |
| 39 | 33.328 | 0.137 | 0.454 | 10.046 | 2.0 | 3.8 | 4.4 | 5.6 | 7.4 | 8.8 | 9.5 | 10.2 | 13.3 | 16.6 | 17.5 | 18.3 | 19.9 | 22.1 | 24.6 | 27.2 |
| 40 | 33.832 | 0.137 | 0.475 | 10.409 | 2.3 | 4.2 | 4.8 | 6.0 | 7.8 | 9.2 | 9.9 | 10.7 | 13.8 | 17.2 | 18.0 | 18.9 | 20.5 | 22.8 | 25.2 | 27.9 |

Supplementary table 4. Week-specific Box-Cox Power Exponential model parameters and selected percentiles of gestational weight gain for pre-pregnancy overweight, Brazilian Maternal and Child Nutrition Consortium data.

| Gestational age (weeks) | Model parameters | | | | Percentiles of gestational weight gain (kg) | | | | | | | | | | | | | | | |
| --- | --- | --- | --- | --- | --- | --- | --- | --- | --- | --- | --- | --- | --- | --- | --- | --- | --- | --- | --- | --- |
|  | mu | sigma | nu | tau | P1 | P2.3 | P3 | P5 | P10 | P16 | P20 | P25 | P50 | P75 | P80 | P84 | P90 | P95 | P97.7 | P99 |
| 10 | 20.111 | 0.190 | 1.072 | 0.822 | -10.9 | -8.3 | -7.5 | -6.0 | -4.0 | -2.7 | -2.1 | -1.5 | 0.1 | 1.7 | 2.3 | 2.9 | 4.2 | 6.1 | 8.4 | 11.0 |
| 11 | 20.424 | 0.189 | 1.071 | 0.850 | -10.7 | -8.1 | -7.3 | -5.8 | -3.8 | -2.5 | -1.9 | -1.3 | 0.4 | 2.1 | 2.7 | 3.3 | 4.6 | 6.6 | 8.8 | 11.4 |
| 12 | 20.742 | 0.189 | 1.070 | 0.878 | -10.5 | -7.9 | -7.1 | -5.6 | -3.6 | -2.2 | -1.6 | -1.0 | 0.7 | 2.5 | 3.1 | 3.7 | 5.0 | 7.0 | 9.3 | 11.7 |
| 13 | 21.066 | 0.189 | 1.070 | 0.907 | -10.2 | -7.7 | -6.9 | -5.4 | -3.3 | -2.0 | -1.4 | -0.8 | 1.1 | 2.9 | 3.5 | 4.1 | 5.4 | 7.4 | 9.7 | 12.1 |
| 14 | 21.394 | 0.188 | 1.069 | 0.937 | -10.0 | -7.5 | -6.7 | -5.2 | -3.1 | -1.8 | -1.1 | -0.5 | 1.4 | 3.3 | 3.9 | 4.5 | 5.9 | 7.9 | 10.1 | 12.5 |
| 15 | 21.729 | 0.188 | 1.069 | 0.967 | -9.8 | -7.3 | -6.5 | -5.0 | -2.9 | -1.5 | -0.9 | -0.2 | 1.7 | 3.7 | 4.3 | 5.0 | 6.3 | 8.3 | 10.5 | 12.9 |
| 16 | 22.069 | 0.188 | 1.068 | 0.998 | -9.5 | -7.0 | -6.3 | -4.7 | -2.7 | -1.3 | -0.6 | 0.0 | 2.1 | 4.1 | 4.7 | 5.4 | 6.7 | 8.7 | 11.0 | 13.4 |
| 17 | 22.415 | 0.187 | 1.068 | 1.029 | -9.3 | -6.8 | -6.0 | -4.5 | -2.4 | -1.0 | -0.4 | 0.3 | 2.4 | 4.5 | 5.2 | 5.8 | 7.2 | 9.2 | 11.4 | 13.8 |
| 18 | 22.767 | 0.187 | 1.067 | 1.061 | -9.0 | -6.6 | -5.8 | -4.3 | -2.2 | -0.8 | -0.1 | 0.6 | 2.8 | 4.9 | 5.6 | 6.3 | 7.7 | 9.7 | 11.9 | 14.2 |
| 19 | 23.126 | 0.186 | 1.067 | 1.094 | -8.7 | -6.3 | -5.5 | -4.0 | -1.9 | -0.5 | 0.2 | 0.9 | 3.1 | 5.4 | 6.0 | 6.7 | 8.1 | 10.1 | 12.3 | 14.6 |
| 20 | 23.490 | 0.186 | 1.066 | 1.128 | -8.4 | -6.0 | -5.3 | -3.8 | -1.7 | -0.2 | 0.5 | 1.2 | 3.5 | 5.8 | 6.5 | 7.2 | 8.6 | 10.6 | 12.8 | 15.1 |
| 21 | 23.861 | 0.186 | 1.065 | 1.163 | -8.1 | -5.8 | -5.0 | -3.5 | -1.4 | 0.1 | 0.8 | 1.5 | 3.9 | 6.2 | 6.9 | 7.6 | 9.1 | 11.1 | 13.3 | 15.5 |
| 22 | 24.238 | 0.185 | 1.065 | 1.198 | -7.8 | -5.5 | -4.7 | -3.2 | -1.1 | 0.3 | 1.1 | 1.8 | 4.2 | 6.7 | 7.4 | 8.1 | 9.5 | 11.6 | 13.7 | 16.0 |
| 23 | 24.621 | 0.185 | 1.064 | 1.234 | -7.5 | -5.2 | -4.5 | -3.0 | -0.9 | 0.6 | 1.4 | 2.1 | 4.6 | 7.1 | 7.9 | 8.6 | 10.0 | 12.1 | 14.2 | 16.4 |
| 24 | 25.009 | 0.185 | 1.064 | 1.269 | -7.2 | -4.9 | -4.2 | -2.7 | -0.6 | 0.9 | 1.7 | 2.4 | 5.0 | 7.6 | 8.3 | 9.1 | 10.5 | 12.6 | 14.7 | 16.9 |
| 25 | 25.404 | 0.184 | 1.063 | 1.305 | -6.9 | -4.6 | -3.9 | -2.4 | -0.3 | 1.2 | 2.0 | 2.7 | 5.4 | 8.1 | 8.8 | 9.6 | 11.0 | 13.1 | 15.2 | 17.4 |
| 26 | 25.805 | 0.184 | 1.063 | 1.339 | -6.6 | -4.3 | -3.6 | -2.1 | 0.0 | 1.5 | 2.3 | 3.1 | 5.8 | 8.5 | 9.3 | 10.1 | 11.5 | 13.6 | 15.7 | 17.8 |
| 27 | 26.211 | 0.184 | 1.062 | 1.373 | -6.3 | -4.0 | -3.3 | -1.8 | 0.3 | 1.8 | 2.6 | 3.4 | 6.2 | 9.0 | 9.8 | 10.6 | 12.0 | 14.1 | 16.2 | 18.4 |
| 28 | 26.623 | 0.183 | 1.062 | 1.404 | -6.0 | -3.7 | -3.0 | -1.5 | 0.6 | 2.2 | 2.9 | 3.7 | 6.6 | 9.5 | 10.3 | 11.1 | 12.6 | 14.6 | 16.7 | 18.9 |
| 29 | 27.042 | 0.183 | 1.061 | 1.434 | -5.7 | -3.4 | -2.7 | -1.2 | 0.9 | 2.5 | 3.3 | 4.1 | 7.0 | 10.0 | 10.8 | 11.6 | 13.1 | 15.1 | 17.3 | 19.4 |
| 30 | 27.467 | 0.182 | 1.060 | 1.462 | -5.3 | -3.1 | -2.4 | -0.9 | 1.2 | 2.8 | 3.6 | 4.5 | 7.5 | 10.5 | 11.3 | 12.1 | 13.6 | 15.7 | 17.8 | 19.9 |
| 31 | 27.898 | 0.182 | 1.060 | 1.487 | -5.0 | -2.8 | -2.1 | -0.6 | 1.6 | 3.2 | 4.0 | 4.8 | 7.9 | 11.0 | 11.8 | 12.6 | 14.1 | 16.2 | 18.4 | 20.5 |
| 32 | 28.336 | 0.182 | 1.059 | 1.510 | -4.7 | -2.5 | -1.7 | -0.3 | 1.9 | 3.5 | 4.3 | 5.2 | 8.3 | 11.5 | 12.3 | 13.1 | 14.7 | 16.8 | 18.9 | 21.0 |
| 33 | 28.780 | 0.181 | 1.059 | 1.530 | -4.4 | -2.2 | -1.4 | 0.1 | 2.3 | 3.9 | 4.7 | 5.6 | 8.8 | 12.0 | 12.8 | 13.6 | 15.2 | 17.3 | 19.5 | 21.6 |
| 34 | 29.232 | 0.181 | 1.058 | 1.547 | -4.1 | -1.9 | -1.1 | 0.4 | 2.6 | 4.3 | 5.1 | 6.0 | 9.2 | 12.5 | 13.4 | 14.2 | 15.8 | 17.9 | 20.1 | 22.2 |
| 35 | 29.690 | 0.181 | 1.058 | 1.562 | -3.8 | -1.5 | -0.8 | 0.7 | 3.0 | 4.6 | 5.5 | 6.4 | 9.7 | 13.0 | 13.9 | 14.7 | 16.3 | 18.5 | 20.7 | 22.8 |
| 36 | 30.156 | 0.180 | 1.057 | 1.575 | -3.5 | -1.2 | -0.4 | 1.1 | 3.3 | 5.0 | 5.9 | 6.8 | 10.2 | 13.5 | 14.4 | 15.3 | 16.9 | 19.1 | 21.3 | 23.4 |
| 37 | 30.630 | 0.180 | 1.056 | 1.584 | -3.2 | -0.9 | -0.1 | 1.4 | 3.7 | 5.4 | 6.3 | 7.2 | 10.6 | 14.0 | 15.0 | 15.8 | 17.5 | 19.7 | 21.9 | 24.1 |
| 38 | 31.110 | 0.180 | 1.056 | 1.591 | -2.9 | -0.5 | 0.2 | 1.8 | 4.1 | 5.8 | 6.7 | 7.6 | 11.1 | 14.6 | 15.5 | 16.4 | 18.1 | 20.3 | 22.5 | 24.7 |
| 39 | 31.599 | 0.179 | 1.055 | 1.596 | -2.5 | -0.2 | 0.6 | 2.2 | 4.5 | 6.2 | 7.1 | 8.1 | 11.6 | 15.1 | 16.1 | 16.9 | 18.6 | 20.9 | 23.2 | 25.4 |
| 40 | 32.095 | 0.179 | 1.055 | 1.601 | -2.2 | 0.1 | 0.9 | 2.5 | 4.9 | 6.6 | 7.5 | 8.5 | 12.1 | 15.7 | 16.6 | 17.5 | 19.2 | 21.5 | 23.8 | 26.1 |

Supplementary table 5. Week-specific Box-Cox Power Exponential model parameters and selected percentiles of gestational weight gain for pre-pregnancy obesity, Brazilian Maternal and Child Nutrition Consortium data.

| Gestational age (weeks) | Model parameters | | | | Percentiles of gestational weight gain (kg) | | | | | | | | | | | | | | | |
| --- | --- | --- | --- | --- | --- | --- | --- | --- | --- | --- | --- | --- | --- | --- | --- | --- | --- | --- | --- | --- |
|  | mu | sigma | nu | tau | P1 | P2.3 | P3 | P5 | P10 | P16 | P20 | P25 | P50 | P75 | P80 | P84 | P90 | P95 | P97.7 | P99 |
| 10 | 19.997 | 0.245 | 1.256 | 0.830 | -13.6 | -10.7 | -9.7 | -7.8 | -5.2 | -3.6 | -2.8 | -2.1 | 0.0 | 2.1 | 2.8 | 3.5 | 5.1 | 7.5 | 10.2 | 13.2 |
| 11 | 20.157 | 0.245 | 1.238 | 0.890 | -13.6 | -10.7 | -9.7 | -7.8 | -5.3 | -3.6 | -2.8 | -2.1 | 0.2 | 2.4 | 3.1 | 3.9 | 5.4 | 7.8 | 10.5 | 13.3 |
| 12 | 20.322 | 0.245 | 1.220 | 0.954 | -13.5 | -10.7 | -9.7 | -7.8 | -5.3 | -3.6 | -2.8 | -2.0 | 0.3 | 2.7 | 3.4 | 4.2 | 5.8 | 8.1 | 10.7 | 13.4 |
| 13 | 20.492 | 0.245 | 1.202 | 1.018 | -13.4 | -10.6 | -9.7 | -7.8 | -5.3 | -3.6 | -2.8 | -2.0 | 0.5 | 3.0 | 3.7 | 4.5 | 6.1 | 8.4 | 10.9 | 13.6 |
| 14 | 20.670 | 0.245 | 1.184 | 1.084 | -13.2 | -10.5 | -9.6 | -7.8 | -5.3 | -3.6 | -2.8 | -1.9 | 0.7 | 3.3 | 4.1 | 4.8 | 6.4 | 8.7 | 11.2 | 13.7 |
| 15 | 20.857 | 0.245 | 1.165 | 1.149 | -13.1 | -10.4 | -9.5 | -7.7 | -5.2 | -3.5 | -2.7 | -1.9 | 0.9 | 3.6 | 4.4 | 5.2 | 6.8 | 9.0 | 11.4 | 13.9 |
| 16 | 21.057 | 0.245 | 1.147 | 1.212 | -12.8 | -10.2 | -9.3 | -7.6 | -5.2 | -3.5 | -2.6 | -1.8 | 1.1 | 3.9 | 4.7 | 5.5 | 7.1 | 9.3 | 11.7 | 14.1 |
| 17 | 21.271 | 0.245 | 1.129 | 1.271 | -12.6 | -10.1 | -9.2 | -7.5 | -5.1 | -3.4 | -2.5 | -1.7 | 1.3 | 4.2 | 5.0 | 5.8 | 7.4 | 9.7 | 12.0 | 14.4 |
| 18 | 21.500 | 0.245 | 1.111 | 1.326 | -12.4 | -9.9 | -9.0 | -7.4 | -5.0 | -3.3 | -2.4 | -1.5 | 1.5 | 4.5 | 5.4 | 6.2 | 7.8 | 10.0 | 12.4 | 14.7 |
| 19 | 21.745 | 0.245 | 1.093 | 1.375 | -12.1 | -9.7 | -8.8 | -7.2 | -4.8 | -3.1 | -2.3 | -1.4 | 1.8 | 4.8 | 5.7 | 6.5 | 8.2 | 10.4 | 12.7 | 15.0 |
| 20 | 22.007 | 0.245 | 1.074 | 1.418 | -11.9 | -9.5 | -8.6 | -7.0 | -4.7 | -3.0 | -2.1 | -1.2 | 2.0 | 5.2 | 6.1 | 6.9 | 8.6 | 10.8 | 13.1 | 15.4 |
| 21 | 22.285 | 0.245 | 1.056 | 1.452 | -11.6 | -9.2 | -8.4 | -6.8 | -4.5 | -2.8 | -1.9 | -1.0 | 2.3 | 5.5 | 6.4 | 7.3 | 9.0 | 11.2 | 13.5 | 15.8 |
| 22 | 22.578 | 0.245 | 1.038 | 1.476 | -11.4 | -9.0 | -8.2 | -6.6 | -4.3 | -2.6 | -1.7 | -0.8 | 2.6 | 5.9 | 6.8 | 7.7 | 9.4 | 11.7 | 14.0 | 16.3 |
| 23 | 22.884 | 0.245 | 1.020 | 1.492 | -11.2 | -8.8 | -8.0 | -6.4 | -4.1 | -2.3 | -1.4 | -0.5 | 2.9 | 6.3 | 7.2 | 8.1 | 9.8 | 12.1 | 14.5 | 16.8 |
| 24 | 23.201 | 0.245 | 1.002 | 1.499 | -10.9 | -8.6 | -7.8 | -6.2 | -3.8 | -2.1 | -1.2 | -0.3 | 3.2 | 6.7 | 7.6 | 8.5 | 10.2 | 12.6 | 15.0 | 17.4 |
| 25 | 23.527 | 0.245 | 0.984 | 1.497 | -10.7 | -8.4 | -7.6 | -6.0 | -3.6 | -1.8 | -0.9 | 0.0 | 3.5 | 7.0 | 8.0 | 8.9 | 10.7 | 13.1 | 15.6 | 18.0 |
| 26 | 23.860 | 0.245 | 0.965 | 1.487 | -10.5 | -8.2 | -7.3 | -5.7 | -3.3 | -1.5 | -0.6 | 0.3 | 3.9 | 7.4 | 8.4 | 9.3 | 11.1 | 13.6 | 16.1 | 18.6 |
| 27 | 24.198 | 0.245 | 0.947 | 1.472 | -10.3 | -7.9 | -7.1 | -5.5 | -3.1 | -1.3 | -0.3 | 0.6 | 4.2 | 7.8 | 8.8 | 9.7 | 11.6 | 14.1 | 16.7 | 19.3 |
| 28 | 24.539 | 0.245 | 0.929 | 1.455 | -10.2 | -7.7 | -6.9 | -5.2 | -2.8 | -1.0 | 0.0 | 1.0 | 4.5 | 8.2 | 9.2 | 10.1 | 12.0 | 14.6 | 17.3 | 20.0 |
| 29 | 24.883 | 0.245 | 0.911 | 1.437 | -10.0 | -7.5 | -6.7 | -5.0 | -2.5 | -0.7 | 0.3 | 1.3 | 4.9 | 8.5 | 9.6 | 10.5 | 12.5 | 15.1 | 17.9 | 20.7 |
| 30 | 25.228 | 0.245 | 0.893 | 1.420 | -9.8 | -7.3 | -6.4 | -4.7 | -2.2 | -0.4 | 0.6 | 1.6 | 5.2 | 8.9 | 10.0 | 11.0 | 12.9 | 15.7 | 18.5 | 21.4 |
| 31 | 25.575 | 0.245 | 0.874 | 1.406 | -9.6 | -7.1 | -6.2 | -4.5 | -2.0 | -0.1 | 0.9 | 1.9 | 5.6 | 9.3 | 10.4 | 11.4 | 13.4 | 16.2 | 19.1 | 22.1 |
| 32 | 25.925 | 0.245 | 0.856 | 1.394 | -9.4 | -6.8 | -6.0 | -4.2 | -1.7 | 0.2 | 1.2 | 2.2 | 5.9 | 9.7 | 10.8 | 11.8 | 13.9 | 16.7 | 19.7 | 22.8 |
| 33 | 26.279 | 0.245 | 0.838 | 1.385 | -9.2 | -6.6 | -5.7 | -4.0 | -1.4 | 0.5 | 1.5 | 2.6 | 6.3 | 10.1 | 11.2 | 12.2 | 14.3 | 17.3 | 20.3 | 23.5 |
| 34 | 26.638 | 0.246 | 0.820 | 1.379 | -8.9 | -6.4 | -5.5 | -3.7 | -1.1 | 0.8 | 1.8 | 2.9 | 6.6 | 10.5 | 11.6 | 12.7 | 14.8 | 17.8 | 21.0 | 24.2 |
| 35 | 27.003 | 0.246 | 0.802 | 1.377 | -8.7 | -6.1 | -5.2 | -3.5 | -0.8 | 1.1 | 2.1 | 3.2 | 7.0 | 10.9 | 12.1 | 13.1 | 15.3 | 18.4 | 21.6 | 24.9 |
| 36 | 27.374 | 0.246 | 0.784 | 1.379 | -8.4 | -5.9 | -5.0 | -3.2 | -0.6 | 1.4 | 2.4 | 3.5 | 7.4 | 11.4 | 12.5 | 13.6 | 15.8 | 18.9 | 22.2 | 25.6 |
| 37 | 27.751 | 0.246 | 0.765 | 1.386 | -8.2 | -5.6 | -4.7 | -2.9 | -0.3 | 1.7 | 2.8 | 3.8 | 7.8 | 11.8 | 13.0 | 14.1 | 16.4 | 19.5 | 22.9 | 26.3 |
| 38 | 28.132 | 0.246 | 0.747 | 1.396 | -7.9 | -5.3 | -4.4 | -2.7 | 0.0 | 2.0 | 3.1 | 4.1 | 8.1 | 12.3 | 13.5 | 14.6 | 16.9 | 20.1 | 23.5 | 27.0 |
| 39 | 28.516 | 0.246 | 0.729 | 1.408 | -7.6 | -5.0 | -4.2 | -2.4 | 0.3 | 2.3 | 3.4 | 4.5 | 8.5 | 12.7 | 13.9 | 15.1 | 17.4 | 20.7 | 24.2 | 27.7 |
| 40 | 28.905 | 0.246 | 0.711 | 1.421 | -7.3 | -4.8 | -3.9 | -2.1 | 0.6 | 2.6 | 3.7 | 4.8 | 8.9 | 13.2 | 14.4 | 15.6 | 18.0 | 21.3 | 24.8 | 28.4 |
