# Supplementary material for: Gestational weight gain charts: results from the Brazilian Maternal and Child Nutrition Consortium
Source: Am J Clin Nutr. 2021 Mar 19;113(5):1351–60. doi: 10.1093/ajcn/nqaa402 (PMC8106749; doi:10.1093/ajcn/nqaa402)

**ONLINE SUPPLEMENTARY MATERIAL**

**Gestational weight gain charts: Results from the Brazilian Maternal and Child Nutrition Consortium**

First authors: Gilberto Kac, Thais R. B. Carrilho

**SUPPLEMENTARY FIGURE**

**FIGURE TITLE AND LEGEND**

Supplementary figure 1. Sample size for the external validation study using Birth in Brazil data.

Note: ‘measurements’ refer to information of both gestational age and weight gain; Underweight, BMI < 18.5 kg/m^2^; Normal, BMI ≥ 18.5 and < 25.0 kg/m^2^; Overweight, BMI ≥ 25.0 and < 30.0 kg/m^2^ and Obesity, BMI ≥ 30.0 kg/m^2^.


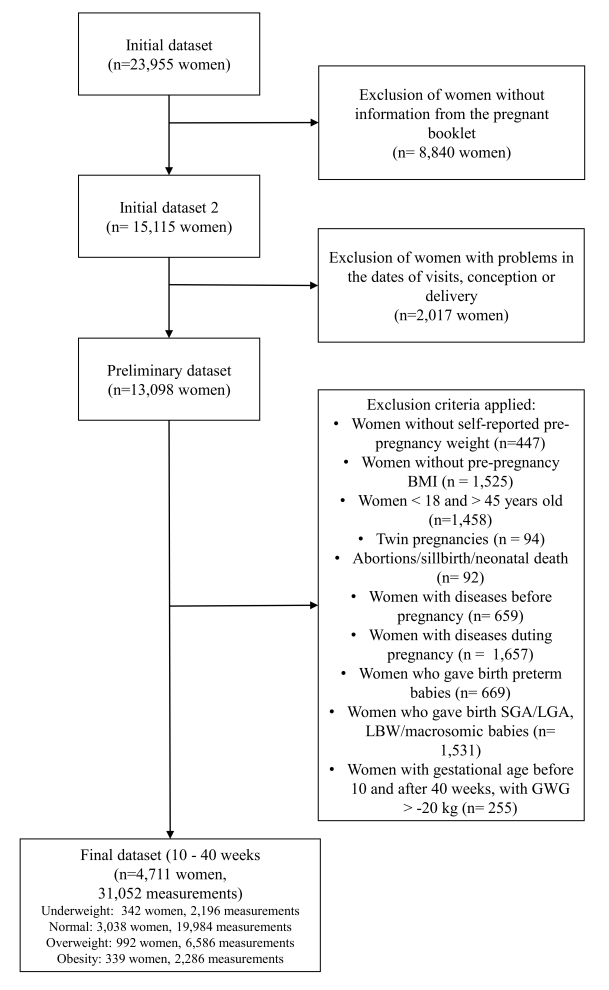

Supplement: nqaa402_Supplemental_Files [file nqaa402_supplemental_files.zip › Sup_fig1_AJCN_27-10-20.docx]
