# Supplementary material for: Gestational weight gain charts: results from the Brazilian Maternal and Child Nutrition Consortium
Source: Am J Clin Nutr. 2021 Mar 19;113(5):1351–60. doi: 10.1093/ajcn/nqaa402 (PMC8106749; doi:10.1093/ajcn/nqaa402)
Supplement: nqaa402_Supplemental_Files [file nqaa402_supplemental_files.zip › Sup_methods1and2_AJCN_10-11-20.docx]

**ONLINE SUPPLEMENTARY MATERIAL**

**Gestational weight gain charts: Results from the Brazilian Maternal and Child Nutrition Consortium**

First authors: Gilberto Kac, Thais R. B. Carrilho

**SUPPLEMENTARY METHODS**

**Supplementary methods 1.** Steps to construct the gestational weight gain charts

All the following steps were performed stratifying according pre-pregnancy BMI category.

**Step 0.** Constructing histograms for GWG in each gestational age (10-40 weeks) to understand the distribution of GWG during pregnancy according to each pre-pregnancy body mass index (BMI) category (R code 1).

**Step 1. Adjusting linear mixed models and extracting percentiles and z-scores.** This is a very naïve approach. The aim was to keep the models as simple as possible. However, the diagnostic of the models (graph of residuals *vs.* fitted values) and the plotting of the percentiles revealed the presence of heteroscedasticity. Based on those results, linear mixed models were found to be inappropriate, as they only model the mean according to gestational age. In cases where heteroscedasticity is observed, modeling the standard deviation (SD) is necessary (Stata code 1).

**Step 2. Adjusting fractional polynomials (FP) and extracting percentiles and z-scores.** This model was performed in two ways: using clusters of individuals and without clusters. The first approach attempted to incorporate the intra-individual variance, which exists because of the repetition of weight measurements for some women in our sample. With FP, it is possible to model both the mean and the SD according to gestational age, providing a solution for the heteroscedasticity problem (1). The incorporation of clusters did not affect percentiles estimation, as they interfere only with the standard error, which is not used in the calculation of the percentiles. Thus, considering the clusters did not change the estimation of the percentiles, which are the values of interest, in our case. The problem of the FP models was the ‘internal validation’. When we compared the percentages of observations above or below some selected percentiles (3/97, 10/90, 25/75, 50) or z-scores (-2/2,-3/3), the values were different from what one should expect, especially in the extreme percentiles (Stata code 2). For instance, in the 3/97 percentiles, one would expect that 3% of the sample would be above the 97^th^ and below the 3^rd^ percentile. In our case, for normal weight women, when FP models were adjusted, 4.8% of the sample were above the 97^th^ centile. According to Cole (2), this is an indication of bias of the models.

**Step 3. Adjusting random effects models modeling gestational age using restricted cubic splines.** This approach allowed us to model weight gain as a function of gestational age using a flexible non-linear model (in each specified knot) (3). A random effects model with unstructured covariance matrix was adjusted, with several knots (3, 4, 5). In those models, the *k* knots are introduced on the *x*-axis (in this case, *x* = gestational age) located at t_1_, t_2_, …, t*_k_*. A model of the expected value of weight gain (*y*) given the gestational age (*x*) is selected, that is linear before t_1_ and after t*_k_*, consists of piecewise cubic polynomials between adjacent knots and is continuous and smooth in each knot (3). This way, it is possible to account for non-linear relations between weight gain and intervals of gestational age. This approach is the same applied by Hutcheon *et al.* (4) and Huang *et al.* (5) when constructing GWG charts for the USA and China, respectively (Stata code 3). The same challenges mentioned on step 2 were present here, i.e., the performance of the model regarding the internal validation was poor even with 5 knots. Increasing the number of knots could lead to overfitting (6), so we decided not to use those models.

**Step 4. Adjusting a combination of FP and multilevel models (ML).** These types of models are similar to those adjusted by Ohuma & Altman (7) for head circumference data, and by Cheikh-Ismail *et al.* (8) for GWG. The use of these models requires the determination of the best-fitting powers for gestational age by modelling GWG as a function of GA using FP. In our case, the best powers were provided by a 2^nd^ order FP for all BMI categories. The functional form of GA was then incorporated to a two-level (individuals and visit) random intercept and slope model. In this model, both mean and SD vary according to GA. However, although it is possible to obtain an equation of the mean, it is not possible to retrieve the equation for the SD, and both are necessary in the calculation of the percentiles. So, after the adjustment of the ML model, it is necessary to model the SD according to GA by using another FP model, to emulate the SD from the ML and to obtain an equation for it. We decided to model the log(SD) to stabilize variance, in the same way as performed by Ohuma & Altman (7) (Stata code 4). Those models should be the best approach for our data, since there are women with repeated GWG measurements, and they provide more accurate equations for the mean and SD. Unfortunately, when we performed the internal validation, by comparing the percentages of observations above or below some selected percentiles (3/97, 10/90, 25/75, 50) or z-scores (-2/2;-3/3), the values were, again, different from the expected, especially in the most extreme percentiles. Besides bias, as mentioned by Cole (2), we considered that the modelling of those percentiles could be affected by kurtosis (9), and none of the models performed could account for that.

**Step 5.** **Adjusting the GAMLSS models.** These models are the same used by the World Health Organization when constructing the growth charts for children (9). By the time of the construction of the charts, a team of experts was consulted and reviewed several models available to construct those types of charts and they concluded in favor of using GAMLSS even with repeated measures, which are not accounted for in those models (10). However, the incorporation of the intra-individual variance in the models would affect the estimation of standard errors of the point estimates of the model, which are not used in the determination of percentiles and z-scores (9).

In our dataset, several attempts were made to find the best distribution, smoother and degrees of freedom for each parameter (mean, deviation, skewness and kurtosis, mu, sigma, nu and tau, respectively) being modeled (all the options are listed in (11)). To avoid the infinite possibilities of tests of specifications of the models, the ‘LMS’ function was used. This function tests and selects the best model from LMS (lambda, mu, sigma, the method proposed by Cole & Green (12)), and besides mean and SD, includes skewness in the modelling and models the parameters using a Box-Cox Cole Green distribution. This function also tests LMST (a modification of LMS that also models kurtosis and used Box-Cox-*t* as distribution) and LMSP (a modification of the LMS that also models kurtosis and used Box-Cox power exponential as distribution). By using this function, the adjustment of the percentiles improved substantially, and the diagnostic revealed very well-adjusted models. Details regarding the implementation of the GAMLSS models are described in the ‘methods’ section (R code 2).

**REFERENCES**

1. Royston P, Altman DG. Regression Using Fractional Polynomials of Continuous Covariates: Parsimonious Parametric Modelling. Journal of the Royal Statistical Society Series C (Applied Statistics) 1994;43(3):429-67. doi: 10.2307/2986270.

2. Cole TJ. Commentary: Methods for calculating growth trajectories and constructing growth centiles. Statistics in medicine 2019;38(19):3571-9. doi: 10.1002/sim.8129.

3. Harrell FE, Jr., Lee KL, Pollock BG. Regression models in clinical studies: determining relationships between predictors and response. . Journal of the National Cancer Institute 1988;80(15):1198-202. doi: 10.1093/jnci/80.15.1198.

4. Hutcheon JA, Platt RW, Abrams B, Himes KP, Simhan HN, Bodnar LM. Pregnancy weight gain charts for obese and overweight women. Obesity 2015;23(3):532-5. doi: 10.1002/oby.21011.

5. Huang A, Xiao Y, Hu H, Zhao W, Yang Q, Ma W, Wang L. Gestational weight gain charts by gestational age and body mass index for Chinese women: A population-based follow-up study. Journal of Epidemiology 2019. doi: 10.2188/jea.JE20180238.

6. Perperoglou A, Sauerbrei W, Abrahamowicz M, Schmid M. A review of spline function procedures in R. BMC Medical Research Methodology 2019;19(1):46. doi: 10.1186/s12874-019-0666-3.

7. Ohuma EO, Altman DG, International F, Newborn Growth Consortium for the 21st C. Statistical methodology for constructing gestational age-related charts using cross-sectional and longitudinal data: The INTERGROWTH-21(st) project as a case study. Statistics in medicine 2019;38(19):3507-26. doi: 10.1002/sim.8018.

8. Cheikh Ismail L, Bishop DC, Pang R, Ohuma EO, Kac G, Abrams B, Rasmussen K, Barros FC, Hirst JE, Lambert A, et al. Gestational weight gain standards based on women enrolled in the Fetal Growth Longitudinal Study of the INTERGROWTH-21st Project: a prospective longitudinal cohort study. BMJ 2016;352:i555. doi: 10.1136/bmj.i555.

9. Borghi E, de Onis M, Garza C, Van den Broeck J, Frongillo EA, Grummer-Strawn L, Van Buuren S, Pan H, Molinari L, Martorell R, et al. Construction of the World Health Organization child growth standards: selection of methods for attained growth curves. Statistics in Medicine 2006;25(2):247-65. doi: 10.1002/sim.2227.

10. Rigby RA, Stasinopoulos DM. Generalized additive models for location, scale and shape. Journal of the Royal Statistical Society: Series C (Applied Statistics) 2005;54(3):507-54. doi: 10.1111/j.1467-9876.2005.00510.x.

11. Stasinopoulos DM, Rigby RA. Generalized Additive Models for Location Scale and Shape (GAMLSS) in R. Journal of Statistical Software 2007;23(7):46. doi: 10.18637/jss.v023.i07.

12. Cole TJ, Green PJ. Smoothing reference centile curves: the LMS method and penalized likelihood. Statistics in Medicine 1992;11(10):1305-19.

**R and STATA codes used for the analysis**

For all codes, an example with normal weight women is provided. In all cases, the models were restricted to 10-40 weeks, due to availability of data.

**Step 0. R code 1**

| # Read The dataset – only normal weight women  library(ggplots2)  nwdata$GA=round(nwdata$gawk_)  nwdata$GA=as.factor(nwdata$GA)  ggplot(nwdata, aes(x=gain_, fill=GA)) +  geom_histogram(binwidth=5) +  facet_wrap(GA~.) +  theme_bw() |
| --- |

**Step 1. Stata code 1 (Based on codes gently provided by Dr. Eric Ohuma)**

| * Read the dataset (Normal weight women only)  * Fit the model for each pre-pregnancy BMI, using xtmixed  xtmixed gain_ gawk_\|\| id: gawk_  * Evaluating residuals – looking for heteroscedasticity:  predict fit1, fitted  predict residuals,rstandard  twoway (scatter residuals fit1)  *Extracting z-scores and percentiles  *Calculating the estimated mean:  predict p_mean, xb  *Calculating the estimated variance:  estat recov  matrix mymatrix=r(cov)  matrix list mymatrix  local var_slope=mymatrix[1,1]  display `var_slope'  local var_cons=mymatrix[2,2]  display `var_cons'  local cov=mymatrix[2,1]  display `cov'  *local var_resfgls_no= (exp(2 * [lnsig_e]_cons))  local var_resfgls_no= 2 * [lnsig_e]_cons  display `var_resfgls_no'  gen p_var = `var_cons' + (`var_slope')*gawk_^2 + 2*gawk_*`cov'+`var_resfgls_no'  label var p_var "Variance"  gen p_sd=sqrt(p_var)  * Generating the percentiles  *P3/97  gen p97=p_mean+1.88*p_sd  gen p3=p_mean-1.88*p_sd  *P5/95  gen p5=p_mean-1.645*p_sd  gen p95=p_mean+1.645*p_sd  *P10/90  gen p10=p_mean-1.28*p_sd  gen p90=p_mean+1.28*p_sd  tabstat p97 p3, s(min max mean)  tabstat p95 p5, s(min max mean)  tabstat p90 p10, s(min max mean)  * Graphing the percentiles  twoway (scatter gain_ gawk_, ms(Oh) mc(gs10)) ///  (line p_mean p3 p10 p90 p97 gawk_, lcolor(red green blue blue green) ///  lwidth(thin thin thin thin thin) sort lpattern(dash dash dash dash dash)), ///  ylabel(-10(10)30) xlabel(10(2)40) ///  scheme(s1mono) plotregion(style(none)) yscale(r(-10 40)) ///  xscale(r(0 40)) xtitle(Gestational age in weeks) ytitle("GWG (Kg)") legend(off) |
| --- |

**Step 2. Stata code 2 (Based on codes gently provided by Dr. Eric Ohuma and Dr. Michael Reichenheim)**

| * Read the dataset (Normal weight women only)  * Fit the model for each pre-pregnancy BMI  * A) FP without clusters  *Identifying the best FP:  xrigls gain_ gawk_, detail nogr  *Best FP Model: m:df 0 0,s:df 2  xrigls gain_ gawk_, fp(m: 0 0,s: 2) centile(3 10 50 90 97) detail nogr  * B) FP with clusters:  * TOGGLE  global clus "ropts(m:vce(cluster id), s:vce(cluster id))"  *Identifying the best FP:  xrigls gain_ gawk_, detail nogr ${clus}  *Best FP Model: m:df 0 0,s:df 2  xrigls gain_ gawk_, fp(m: 0 0,s: 2) centile(3 10 50 90 97) detail nogr ${clus}  **** Procedures to be adopted for both approaches:  *Generating z-scores and centiles:  foreach var of varlist C3_gls C50_gls C97_gls C10_gls C90_gls C25_gls C75_gls Z_gls {  gen `var'_no=`var'  }  *Plotting centiles  set more on  #delimit ;  twoway (scatter gain_ gawk_, ms(Oh) mc(gs10) )  (line C3_gls_no C50_gls_no C97_gls_no gawk_, lcolor(blue blue blue)  sort lpattern(dash dash dash)), ylabel(-10(5)40) xlabel(10(2)40)  scheme(s1mono) plotregion(style(none)) ysca(titlegap(*6))  xsca(titlegap(*6)) xtitle(Gestational age in weeks) ytitle("GWG (Kg)") legend(off)  ;  #delimit cr  *Internal validation  gen complete_weeks=int(gawk_)  ** Number of obs below 3^rd^ or above 97^th^ centiles  gen below_c3 = cond(gain_<C3_gls,1,0)  tab below_c3  bysort complete_weeks: tab below_c3    gen above_c97 = cond(gain_> C97_gls,1,0)  tab above_c97  bysort complete_weeks: tab above_c97    ** Number of obs below 10^th^ or above 90^th^ centiles  gen below_c10 = cond(gain_< C10_gls ,1,0)  tab below_c10  bysort complete_weeks: tab below_c10    gen above_c90 = cond(gain_> C90_gls,1,0)  tab above_c90  bysort complete_weeks: tab above_c90  ** Number of obs below 25^th^ or above 75^th^ centiles  gen below_c25 = cond(gain_< C25_gls ,1,0)  tab below_c25  bysort complete_weeks: tab below_c25    gen above_c75 = cond(gain_> C75_gls,1,0)  tab above_c75  bysort complete_weeks: tab above_c75 |
| --- |

**Step 3. Stata code 3 (Based on codes gently provided by Dr. Eric Ohuma and Dr. Jennifer Hutcheon)**

| * Read the dataset (Normal weight women only)  * Fit the model for each pre-pregnancy BMI  *Log-transforming the weight gain variable  *Adding a constant to the weight gain variable so that there are no negative values (which can't be log-transformed)  sum gain_  hist gain_  gen gwg_=gain+25  gen logweight_gain_cumulative=log(gwg_)  sum logweight_gain_cumulative  *Creating a spline for gestational age (in weeks)  * Compare models with different knots to identify the best one  rc_spline gawk_, nknots(3)  *Random intercept and random slope model, unstructured covariance  xtmixed logweight_gain_cumulative _S*\|\| id: _Sgawk_1, cov(unstr) mle variance  estat ic  drop _S*  rc_spline gawk_, nknots(4)  xtmixed logweight_gain_cumulative _S*\|\| id: _Sgawk_1, cov(unstr) mle variance  estat ic  drop _S*  rc_spline gawk_, nknots(5)  xtmixed logweight_gain_cumulative _S*\|\| id: _Sgawk_1, cov(unstr) mle variance  estat ic  drop _S*  *Consider the model with lowest BIC and AIC and run it again  * Final model  rc_spline gawk_, nknots(5)  *Random intercept and random slope model, unstructured covariance  xtmixed logweight_gain_cumulative _S*\|\| id: _Sgawk_1, cov(unstr) mle variance  estat ic  * Calculating the estimated mean:  predict p_mean, xb  *Calculating the estimated variance:  estat recov  matrix mymatrix=r(cov)  matrix list mymatrix  local var_slope=mymatrix[1,1]  display `var_slope'  local var_cons=mymatrix[2,2]  display `var_cons'  local cov=mymatrix[2,1]  display `cov'  local var_resfgls_no= (exp(2 * [lnsig_e]_cons))  gen p_var = `var_cons' + (`var_slope')*_Sgawk_1^2 + 2*_Sgawk_1*`cov'+`var_resfgls_no'  label var p_var "Variance"  gen p_sd=sqrt(p_var)  * Back-converting to unstransformed scale  gen exp_p_mean = exp(p_mean) - 25  gen C50_rcs = exp(p_mean) - 25  * Obtaining SDs TO PLOT  *i.e., to obtain 1 SD  gen exp_lower_sd=(exp(p_mean-1*sqrt(p_var))) - 25  gen exp_upper_sd=(exp(p_mean+1*sqrt(p_var))) - 25  * Graphing mean and SD  twoway (scatter gain_ gawk_, msymbol(oh) mcolor(gs10)) ///  (connected exp_p_mean gawk_, msymbol(none) lcolor(blue) lwidth(medthick) lpattern(solfgls_no)) ///  (connected exp_lower_sd gawk_, msymbol(none) lcolor(red) lwidth(medthick) lpattern(dash)) ///  (connected exp_upper_sd gawk_, msymbol(none) lcolor(red) lwidth(medthick) lpattern(dash)), legend(off) ///  ytitle("Gestational weight gain (kg)") xtitle("Gestational age (weeks)") scale(1.35) ylabel(, nogrid) graphregion(color(white)) ///  xlabel(10(2)40) xsca(titlegap(*6)) ysca(titlegap(*6))  * Perform internal validation as before |
| --- |

**Step 4. Stata code 4 (Based on codes gently provided by Dr. Eric Ohuma)**

| * Read the dataset (Normal weight women only)  * Fit the model for each pre-pregnancy BMI  * Identifying the best FP  xrigls gain_ gawk_, detail nogr  ** Best model: Powers for the mean: -1, 0.5; for the SD 1.0  xrigls gain_ gawk_, fp(m: -1 0.5,s: 1) centile(3 10 50 90 97) detail  ** Multi-level models  ** 1) using the best FP powers (-1,0.5)  global MLwiN_path C:\Program Files (x86)\MLwiN trial\i386\mlwin.exe  bysort origin id (gawk_): gen occasion = _n  tab occasion  format gain_ %9.3f  gen cons=1  * Creating variables according to the FP model  gen gw1 = (gawk_)^-1  gen gw2 = (gawk_)^0.5  cap drop u0 u1 u2 u0se u1se u2se  sort id occasion  runmlwin gain_ cons gw1 gw2, level2(id: cons gw1 gw2,residuals(u)) level1(occasion: cons) maxiterations(1000) nopause rigls  est store rs_2levels  estimates table rs_2levels, stats(N deviance ll) b(%4.3f) stfmt(%4.0f) varwidth(18)  * Predict the average gwg for the average subject  predict rsmean_2levels, xb  * Add the subject residuals onto the predictions for the average gwg line  generate rsmean_2levelsxbu = rsmean_2levels + u0 + u1*gw1 + u2*gw2  * Sort the data by id and then by gawk_ within each subject  sort id gawk_  * Plot the predicted subject lines for GWG  twoway (line rsmean_2levelsxbu gawk_, connect(ascending)), ///  ytitle("Predicted GWG") xtitle("Gestational age (weeks)") ///  title(RS model (2-levels)) ///  scheme(s1color) plotregion(style(none)) xsize(20) ysize(18) ///  ylabel(-20(5)35) xlabel(10 (4) 40) ysca(titlegap(*10)) ///  xsca(titlegap(*6))  * Predict the level 2 variance function  generate rslev2var = ///  [RP2]var(cons) ///  + 2*[RP2]cov(cons\gw1)*gw1 + [RP2]var(gw1)*gw1^2 ///  + 2*[RP2]cov(cons\gw2)*gw2 + 2*[RP2]cov(gw1\gw2)*gw1*gw2 + [RP2]var(gw2)*gw2^2    generate rslev2sd = sqrt(rslev2var)  * Plot the subject-level variance function  * Observe the variance/sd increase with GA  line rslev2var gawk_, sort xlabel(10 (2) 40) xtitle(Gestational age (weeks)) ///  ytitle("Between-subject variance") title("RS (2-levels) variance by GA") scheme(s1color) ///  plotregion(style(none)) legend(off) ysca(titlegap(*10)) xsca(titlegap(*6)) xsize(20) ysize(18)  line rslev2sd gawk_, sort xlabel(10 (2) 40) xtitle(Gestational age (weeks)) ///  ytitle("Between-subject variability (SD)") title("RS (2-levels) in SD") ///  scheme(s1color) plotregion(style(none)) legend(off) ysca(titlegap(*10)) xsca(titlegap(*6)) xsize(20) ysize(18)  * Generate the predicted 97 and 3 centiles  generate rslev2high97 = rsmean_2levels + 1.88*rslev2sd  generate rslev2low3 = rsmean_2levels - 1.88*rslev2sd  * Generate the predicted 90 and 10 centiles  generate rslev2high90 = rsmean_2levels + 1.28*rslev2sd  generate rslev2low10 = rsmean_2levels - 1.28*rslev2sd  * Generate the predicted 1SD  generate rslev2high1SD = rsmean_2levels + 1*rslev2sd  generate rslev2low1SD = rsmean_2levels - 1*rslev2sd  * Generate the predicted 2SD  generate rslev2high2SD = rsmean_2levels + 2*rslev2sd  generate rslev2low2SD = rsmean_2levels - 2*rslev2sd  * Plotting percentiles  format gain_ %9.0f  * Plot the predicted mean relationship together with the predicted centiles  twoway (scatter gain_ gawk_ if ppns==0,msymbol(smcircle_hollow) mcolor(gs12)) ///  (line rslev2low3 rsmean_2levels rslev2high97 gawk_ if ppns==0, sort lcolor(red red red) lpattern(dash dash dash)), ///  ylabel(-10(5)30) xlabel(10 (2) 40) xtitle(Gestational age (weeks)) ytitle("GWG (kg)") ///  title("Random Slope model (2-levels)") scheme(s1color) plotregion(style(none)) legend(col(4) ///  order(1 4 ) lab(1 "Raw data") lab(4 "RS (2-levels)")) ysca(titlegap(*10)) ///  xsca(titlegap(*6)) xsize(20) ysize(18)  * Plot the predicted mean relationship together with the predicted centiles  twoway (scatter gain_ gawk_ if ppns==0,msymbol(smcircle_hollow) mcolor(gs12)) ///  (line rslev2low3 rslev2high97 gawk_ if ppns==0, sort lcolor(blue blue) lpattern(dash dash dash)) ///  (line rslev2low10 rsmean_2levels rslev2high90 gawk_ if ppns==0, sort lcolor(red green red) lpattern(dash dash dash)), ///  ylabel(-10(5)30) xlabel(10 (2) 40) xtitle(Gestational age (weeks)) ytitle("GWG (kg)") ///  title("Random Slope model (2-levels)") scheme(s1color) plotregion(style(none)) legend(col(4) ///  order(1 4 ) lab(1 "Raw data") lab(4 "RS (2-levels)")) ysca(titlegap(*10)) ///  xsca(titlegap(*6)) xsize(20) ysize(18)  * Plot the predicted mean relationship together with the predicted z-scores  twoway (scatter gain_ gawk_ if ppns==0,msymbol(smcircle_hollow) mcolor(gs12)) ///  (line rslev2low2SD rslev2high2SD gawk_ if ppns==0, sort lcolor(blue blue) lpattern(dash dash dash)) ///  (line rslev2low1SD rsmean_2levels rslev2high1SD gawk_ if ppns==0, sort lcolor(red green red) lpattern(dash dash dash)), ///  ylabel(-10(5)30) xlabel(10 (2) 40) xtitle(Gestational age (weeks)) ytitle("GWG (kg)") ///  title("Random Slope model (2-levels)") scheme(s1color) plotregion(style(none)) legend(col(4) ///  order(1 4 ) lab(1 "Raw data") lab(4 "RS (2-levels)")) ysca(titlegap(*10)) ///  xsca(titlegap(*6)) xsize(20) ysize(18)    *** Internal validation  gen complete_weeks=int(gawk_)  ** Number of obs below 3^rd^ or above 97^th^ centiles  gen below_c3 = cond(gain_<rslev2low3 ,1,0)  tab below_c3  bysort complete_weeks: tab below_c3    gen above_c97 = cond(gain_>rslev2high97,1,0)  tab above_c97  bysort complete_weeks: tab above_c97    ** Number of obs below 10^th^ or above 90^th^ centiles  gen below_c10 = cond(gain_<rslev2low10 ,1,0)  tab below_c10  bysort complete_weeks: tab below_c10    gen above_c90 = cond(gain_>rslev2high90,1,0)  tab above_c90  bysort complete_weeks: tab above_c90 |
| --- |

**Step 5. R code 2 (Extraction of GAMLSS centiles used a function created by prof. Stef van Buuren and gently provided by Dr. Iris Eekhout)**

| # Read the dataset (Normal weight women only)  # Fit the model for each pre-pregnancy BMI  library(gamlss)  # Adding 20kg to weight gain, since it cannot have negative or 0 values  summary(nwdata$gain_)  nwdata$gwg = nwdata$gain_+20  summary(nwdata$gwg)  # Rounding the GA for 2 digits  nwdata$ga=round(nwdata$gawk_, digits=2)  # Using LMS function  m1 <- lms(y=gwg, x=ga, data= nwdata, trans.x=F, n.cyc = 20)  # Extracting the DF  df=cbind(m1$mu.df,m1$sigma.df,m1$nu.df,m1$tau.df)  df  # Diagnostic  plot(m1)  fittedPlot(m1, x=nwdata$ga)  wp(m1, xvar = nwdata$ga, n.inter = 20, ylim.worm=1.0)  Q.stats(m1, xvar = nwdata$ga, n.inter=20)  # Ditribution by GA  library(gamlss.util)  plotSimpleGamlss(gwg,ga,m1, data=nwdata, x.val=seq(10,40,2),xlim=c(-10,40))  # Selected centiles: 3,10,25,50,75,90,97  centiles(m1, xvar=nwdata$ga, cent = c(3, 10, 25, 50, 75, 90, 97),  legend = F, ylab = "Gestational weight gain (kg)", xlab = "Gestational age (weeks)",  main = NULL, main.gsub = NULL, xleg = min(xvar), yleg = max(obj$y), save = FALSE,  plot = TRUE, points = TRUE, pch = 15, cex = 0.5, col = gray(0.75),  col.centiles = c("blue", "darkgreen", "orange", "red", "orange", "darkgreen","blue"), lty.centiles = 1, lwd.centiles = 4)  # Codes adapted from Prof. Van Buuren/Dr. Iris Eekhout  # Extracting values for the table  # Make a new data frame with a column for gestational age and for other covariates if they exist  nd <- data.frame(ga=c(10:40))  # Use the predict function from gamlss to predict the mu, sigma, nu and tau for the new data (nd), according the model (m1)  refpred.BMI0<- predictAll(m1, terms = c("mu", "sigma", "nu", "tau"), newdata = nd, data = nwdata)  ref.fitBMI0 <-data.frame(pop="meta", sex="W",  sub="N",  x=nd[,1],  mu=round(refpred.BMI0$mu,4),  sigma=round(refpred.BMI0$sigma,4),  nu=round(refpred.BMI0$nu,4),  tau=round(refpred.BMI0$tau,4))  # Transform the weight gain variable (gwg) in the data, to a z-score using the reference table.  # In the distribution option, you can specify the model that you used. The reference (ref) should contain a column for each parameter in the model.  # Package AGD is necessary  library (AGD)  nwdata$Zscores<- y2z(y=nwdata$gwg, x=nwdata$ga, sex="W", sub="N", ref=ref.fitBMI0, dist="BCT", dec=4)  ## Function to get the centiles based on the z-scores  get.centiles <- function(  z=z,  x=x,  ref=ref,  sex="W",  sub=sub,  dec=2) {  zr <- rep(z,times=length(x))  xr <- rep(x,each=length(z))  w <- z2y(z=zr,x=xr,sex=sex,sub=sub, ref=ref,dec=dec,dist="BCT")  w <- matrix(w,ncol=length(z), byrow=TRUE)  w <- data.frame(sub=sub,sex=sex,x=x,round(w,dec), row.names=NULL)  dimnames(w)[[2]] <- c("sub", "sex", "x", as.character(z))  return(w)  }  ## Get the centiles to use for the plot  x <- c(10:40)  percentiles <- c(0.01,0.023,0.03,0.05,0.10,0.16,0.20,0.25,0.50,0.75,0.80,0.84,0.90,0.95,0.977,0.99)  z <- qnorm(percentiles) #transform percentiles to z-scores  centile_refs_BMI0 <- get.centiles(x=x, sub="N",z=z,ref=ref.fitBMI0)  centile_refs_BMI0 <- data.frame(bmigr=1,centile_refs_BMI0)  colnames(centile_refs_BMI0) <- c("bmigr", "sub","sex", "x", paste0("p",as.character(percentiles*100)))  # Set the centile refs back to the original scale (0 is no gain)  centile_refs_BMI0[,paste0("p",as.character(percentiles*100))] <- (centile_refs_BMI0[,paste0("p",as.character(percentiles*100))])-20  ## New graph – scale back to zero  g1 <- ggplot(centile_refs_BMI0, aes(x,p50))+geom_line(size=1.2, colour="red")+  geom_line(aes(x,p10), colour="darkgreen", size=1.2)+  geom_line(aes(x,p90), colour="darkgreen", size=1.2)+  geom_line(aes(x,p25),colour="orange", size=1.2)+  geom_line(aes(x,p75), colour="orange", size=1.2)+  scale_x_continuous(breaks=seq(10,40,1), limits=c(10,40))+  scale_y_continuous(breaks=seq(-10,25,2), limits=c(-10,25))+  theme_bw() +  theme(panel.border = element_blank(),  axis.line = element_line(colour = "black"),  panel.grid.major = element_line(),  panel.grid.major.x = element_blank(),  panel.grid.major.y = element_blank(),  panel.grid.minor = element_blank(),  panel.grid.minor.x = element_blank(),  panel.grid.minor.y = element_blank(),  strip.background = element_rect(colour = "black", size = 0.5),  legend.key = element_blank(),  axis.text.y = element_text(size=12, family="serif", color="black"),  axis.text.x = element_text(size=14,family="serif", color="black", hjust = 1, vjust=0.5),  axis.title.x = element_text(size=15,family="serif", color="black",margin=margin(20,0,0,0)),  axis.title.y = element_text(size=15,family="serif", color="black",margin=margin(0,20,0,0))  )+  xlab("Gestational age (weeks)")+ ylab("Weight gain (kg), BMI Normal")  g1  # Saving the parameters and centiles  save(ref.fitBMI0, file="reffitBMI0_10-40.RData")  write.csv2(centile_refs_BMI0, file="centiles_normal10-40.csv") |
| --- |

**Supplementary methods 2. Example of calculation of a woman’s z-score according to the Brazilian gestational weight gain charts** (Generalized Additive Models for Location, Scale and Shape with BCTo and BCPEo were used).

Example: Woman with self-reported pre-pregnancy weight of 60.0 kg and height of 160 cm, has a pre-pregnancy BMI of 23.4 kg/m^2^, and is classified as normal weight. At the 32^nd^ gestational weeks, she has gained 10 kg. This value is used to classify her z-score according to gestational age, using the equation from figure 2 (copied below) and the model parameters from Supplementary table 3. At 32 gestational weeks, M = 29.9311, S = 0.1305, L (or nu) = 0.3089 (hence L ≠ 0), so, using the first equation:


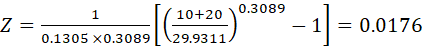

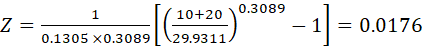


This woman is in the 0.0176 z-score, consequently, around the 50^th^ percentile at the 32^nd^ gestational week.
